# Selective inhibition of JAK3 signaling is sufficient to reverse alopecia areata

**DOI:** 10.1172/jci.insight.142205

**Published:** 2021-04-08

**Authors:** Zhenpeng Dai, James Chen, Yuqian Chang, Angela M. Christiano

**Affiliations:** 1 Department of Dermatology and; 2 Department of Genetics and Development, Columbia University, Vagelos College of Physicians and Surgeons, New York, New York, USA.

**Keywords:** Autoimmunity, Inflammation, Skin, T cells

## Abstract

The Janus kinase/signal transducers and activators of transcription (JAK/STAT) are key intracellular mediators in the signal transduction of many cytokines and growth factors. Common γ chain cytokines and interferon-γ that use the JAK/STAT pathway to induce biological responses have been implicated in the pathogenesis of alopecia areata (AA), a T cell–mediated autoimmune disease of the hair follicle. We previously showed that therapeutic targeting of JAK/STAT pathways using the first-generation JAK1/2 inhibitor, ruxolitinib, and the pan-JAK inhibitor, tofacitinib, was highly effective in the treatment of human AA, as well as prevention and reversal of AA in the C3H/HeJ mouse model. To better define the role of individual JAKs in the pathogenesis of AA, in this study, we tested and compared the efficacy of several next-generation JAK-selective inhibitors in the C3H/HeJ mouse model of AA, using both systemic and topical delivery. We found that JAK1-selective inhibitors as well as JAK3-selective inhibitors robustly induced hair regrowth and decreased AA-associated inflammation, whereas several JAK2-selective inhibitors failed to restore hair growth in treated C3H/HeJ mice with AA. Unlike JAK1, which is broadly expressed in many tissues, JAK3 expression is largely restricted to hematopoietic cells. Our study demonstrates inhibiting JAK3 signaling is sufficient to prevent and reverse disease in the preclinical model of AA.

## Introduction

Alopecia areata (AA) is an autoimmune disease of the hair follicle (HF) that ranges in presentation from circular patches on the scalp to complete hair loss and is associated with an enormous psychological burden to patients (1, 2). The etiology of AA is not completely understood but likely involves a combination of genetic predisposition and environmental triggers (3). We previously showed that cytotoxic natural killer group 2 member D–positive (NKG2D^+^), CD8^+^ T cells accumulate in the skin and contribute to HF destruction (4, 5). The pathogenesis of AA is also associated with the overexpression of proinflammatory cytokines, such as interferon-γ (IFN-γ) and common γ chain (γc) cytokines, which break down HF immune privilege and promote the survival and function of cytotoxic T lymphocytes in affected skin (6, 7). Notably, these proinflammatory cytokines signal through their receptors via the family of Janus kinase/signal transducers and activators of transcription (JAK/STAT).

JAK/STAT pathways play an essential role in both innate and adaptive immunity as well as hematopoiesis. Unrestrained activation of the JAK/STAT pathways contributes to a number of autoimmune diseases and proliferative disorders, making JAKs an attractive target for pharmacologic manipulation in the treatment of such conditions (8, 9). Indeed, small molecule JAK inhibitors (JAKi) demonstrated clinical efficacy in the treatment of rheumatoid arthritis and myelofibrosis, as well as other autoimmune and malignant proliferative disorders (10–12). AA is characterized by dysregulation of JAK/STAT activity, in particular, the γc cytokine and IFN-γ signaling pathway (3, 4). Our lab recently pioneered the use of the JAK1/2 inhibitor ruxolitinib and baricitinib, as well as the pan-JAK inhibitor, tofacitinib, in the treatment of human AA (3, 13–15). However, the relative contribution of JAK1, JAK2, and JAK3 inhibition to the therapeutic benefit of ruxolitinib, baricitinib, and tofacitinib in AA has not been investigated.

Recently, a number of JAK-selective inhibitors have entered clinical trials for the treatment of various malignancies and inflammatory diseases. For example, INCB039110, a JAK1-selective inhibitor, showed efficacy in phase II trials of chronic plaque psoriasis and myelofibrosis (16, 17). The JAK2-selective inhibitor CEP-33779 appeared to be efficacious in mouse models of systemic lupus erythematosus (18). Fedratinib and pacritinib are additional JAK2-selective inhibitors that showed therapeutic efficacy in a murine model of myeloproliferative disease as well as myeloid and lymphoid malignancies, respectively (19, 20). The irreversible covalent JAK3-selective inhibitor PF-06651600 was shown to be effective in rodent models of arthritis and mouse models of multiple sclerosis (21).

Our previous gene expression studies suggested a clear role for JAK1 and JAK3 in AA disease pathogenesis, but notably, not for JAK2 signaling (4, 13–15). Due to the essential role of JAK2 in hematopoiesis, JAK2 inhibition is believed to be the source of several known side effects in other diseases (19). Therefore, highly selective inhibition of JAK1 or JAK3, with no off-target activity against other JAKs, could potentially enhance efficacy and reduce the risk of undesirable side effects. However, it is not known whether selective inhibition of JAK1 or JAK3 alone is sufficient to disrupt AA cytokine signaling and ameliorate the inflammatory processes. The availability of selective JAKi now enables the pharmacological investigation of IFN-γ and γc cytokine signaling, since JAK2-selective inhibitors target IFN-γ signaling, JAK3-selective inhibitors target γc cytokine signaling, and JAK1-selective inhibitors target both cytokine signaling pathways.

To interrogate the role of individual JAKs in AA, we used a panel of JAK-selective inhibitors to treat C3H/HeJ mice with AA. We found that simultaneous IFN-γ and γc cytokine signaling blockade by JAK1-selective inhibitors, as well as inhibition of γc signaling alone with JAK3-selective inhibitors, potently induced hair growth in C3H/HeJ mice with AA. In contrast, JAK2-selective inhibitors failed to restore hair regrowth, indicating that inhibition of IFN-γ alone is not sufficient for treatment of AA in the C3H/HeJ mouse model of AA. Our results establish that γc cytokine signaling pathways are the primary therapeutic targets in AA treatment and that reversal can be achieved with either JAK1 inhibition or JAK3 inhibition.

## Results

### Inhibition of cytokine-dependent signaling by JAK-selective inhibitors.

**Figure 1:**
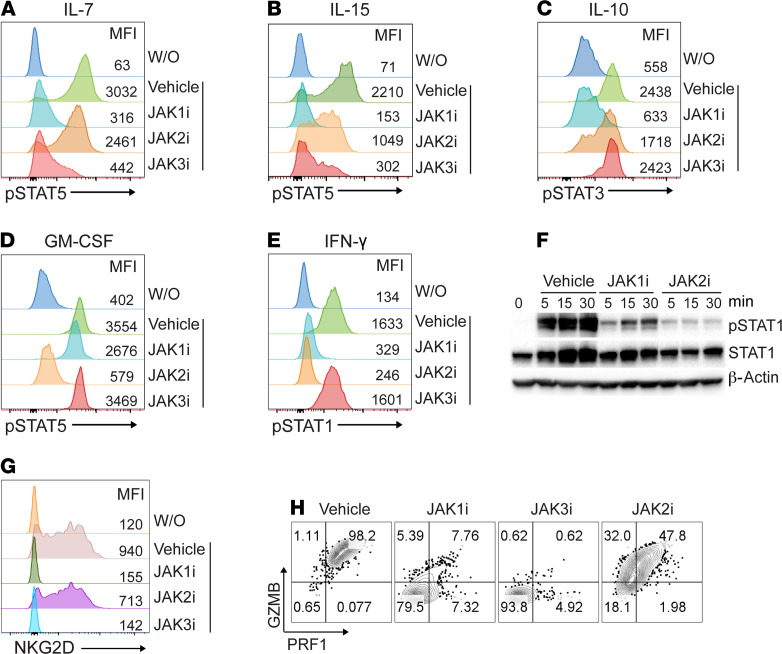
Inhibition of cytokine-dependent signaling by JAK-selective inhibitors. (**A**–**E**) C3H/HeJ mouse splenocytes were pretreated with 1 μM of INCB039110 (JAK1i), CEP-33779 (JAK2i), or PF-06651600 (JAK3i) or vehicle control for 1 hour at 37°C. The treated cells were stimulated with IL-7 (20 ng/mL), IL-15 (40 ng/mL), IL-10 (50 ng/mL), GM-CSF (50 ng/mL), or IFN-γ (50 ng/mL) for 20 minutes at 37°C. Phosphorylated STAT (p-STAT) expression in indicated cell subsets was presented as representative plots and mean fluorescence intensity (MFI). CD3^+^CD8^+^ cells (**A** and **B**), CD19^+^ cells (**C**), CD11b^+^ cells (**D**), and CD3^+^ cells (**E**) were gated for p-STAT expression. (**F**) C3H/HeJ mouse HF dermal sheath cells were pretreated with 1 μM of INCB039110, CEP-33779, or vehicle control for 1 hour at 37°C. The treated cells were then stimulated with IFN-γ (50 ng/mL) for indicated time points at 37°C and analyzed by Western blotting for p-STAT1, total STAT1, and the housekeeping protein β-actin. (**G** and **H**) C3H/HeJ mouse splenocytes were pretreated with 500 nM of indicated JAKi or vehicle control for 1 hour at 37°C. The treated cells from C3H/HeJ mice were then stimulated with 20 ng/mL IL-15 and 250 nM of indicated JAKi at 37°C for 72 hours. (**G**) Expression of NKG2D and (**H**) expression of Granzyme B (GZMB) and Perforin 1 (PRF1) by CD8^+^ T cells presented as representative plots and MFI. The data shown are from 1 representative experiment out of 2 replicates. W/O, without treatment.

To investigate the specificity of JAKi on cytokine-dependent STAT phosphorylation in vitro using different primary mouse cell types, first, we stimulated murine splenocytes pretreated with INCB039110 (JAK1i), CEP-33779 (JAK2i), or PF-06651600 (JAK3i) with various cytokines (Supplemental Table 1; supplemental material available online with this article; https://doi.org/10.1172/jci.insight.142205DS1). We tested IL-7 and IL-15, which signal through JAK1/3 and lead to STAT5 tyrosine phosphorylation, and found that INCB039110 and PF-06651600 (but not CEP-33779) robustly inhibited this response (Figure 1, A and B). Next, we used IL-10 to induce STAT3 tyrosine phosphorylation via JAK1/TYK2 and found that it was specifically inhibited by INCB039110 (Figure 1C). In primary mouse macrophages, we observed that only CEP-33779 (but not INCB039110 or PF-06651600) inhibited JAK2-dependent GM-CSF signaling (Figure 1D). IFN-γ signaling through JAK1/2-induced STAT1 tyrosine phosphorylation, which was inhibited by INCB039110 or CEP-33779 (but not by PF-06651600) (Figure 1E). Finally, in mouse HF dermal sheath cells, we found that INCB039110 or CEP-33779 inhibited IFN-γ–induced STAT1 tyrosine phosphorylation (Figure 1F). Together, these data demonstrated that INCB039110, CEP-33779, and PF-06651600 selectively inhibited their respective JAKs in vitro.

### Induction of NKG2D^+^CD8^+^ T cells was blocked by JAK1- or JAK3-selective inhibitor.

We previously showed that cytotoxic NKG2D^+^CD8^+^ T cells are both necessary and sufficient to induce AA and that IL-15 is a critical γc cytokine for NKG2D^+^CD8^+^ T cell induction in AA (4). To investigate the role of JAK signaling in NKG2D^+^CD8^+^ T cell induction, we stimulated naive CD8^+^ T cells with IL-15 in the presence of individual JAKi. IL-15 robustly induced NKG2D^+^CD8^+^ T cells, and both INCB039110 and PF-06651600 abolished this effect (Figure 1G). IL-15 also augmented the cytotoxic function of CD8^+^ T cells by increasing the production of granzymes and perforin. This effect was markedly reduced in CD8^+^ T cells treated with INCB039110 or PF-06651600 compared with CEP-33779 (Figure 1H). Together, these data demonstrate that JAK1- or JAK3-selective inhibitors selectively inhibited γc cytokine signaling.

### Skin infiltrating CD8^+^ T cells were responsive to γc cytokine stimulation.

Our previous RNA-Seq analysis showed that γc cytokines and their receptors, including IL-7Rα (CD127) and IL-15Rβ (CD122), were upregulated in alopecic skin from both human patients and C3H/HeJ mice with AA (4). We examined the expression of CD122 and CD127 in skin infiltrating CD8^+^ T cells at the protein level. We observed similar levels of CD122 or CD127 on CD8^+^ T cells between lymphoid organs and skin (Supplemental Figure 2A). Furthermore, IL-7 and IL-15 induced comparable levels of STAT5 tyrosine phosphorylation in CD8^+^ T cells between lymphoid organs and skin (Supplemental Figure 2B). These data indicate that skin infiltrating CD8^+^ T cells are likely dependent on γc cytokines for their survival and function.

### Systemic treatment with JAK1-selective inhibitor reversed AA.

**Figure 2:**
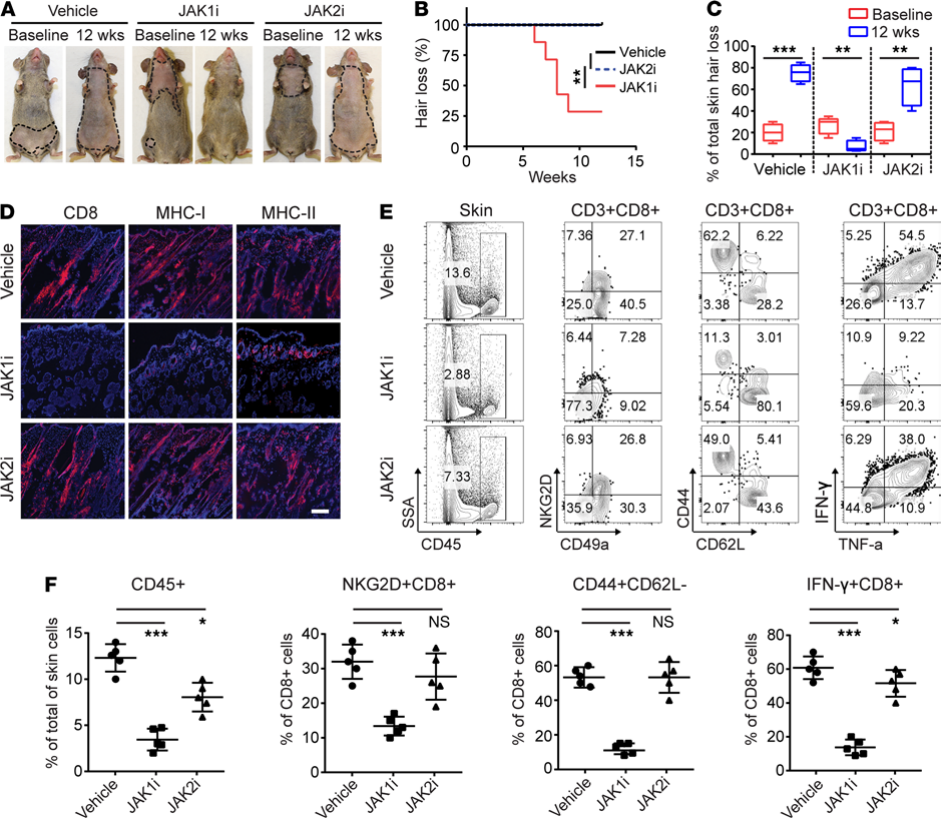
JAK1-selective inhibitor treatment reversed AA. Five C3H/HeJ AA mice per group were treated systemically with INCB039110 (JAK1i) or CEP-33779 (JAK2i) at a dosage of 50 mg/kg for 12 weeks. (**A**) Representative images of individual JAK3i or vehicle-treated C3H/HeJ mice before or after 12 weeks’ treatment. (**B**) Time course of hair regrowth shown as weeks after treatment. **P < 0.01, log-rank test. (**C**) Percentage of total skin hair loss or regrowth shown before and after treatment. The box plots depict the minimum and maximum values (whiskers), the upper and lower quartiles, and the median. The length of the box represents the interquartile range. **P < 0.01, ***P < 0.001 (unpaired Student’s *t* test). (**D**) Representative immunofluorescence images of skin sections from JAKi- or control-treated mice, stained with anti-CD8, anti–MHC class I, or anti–MHC class II mAbs. Scale bar: 200 μm. (**E**) and (**F**) Percentages of skin infiltrating CD45^+^ leukocytes, CD44^+^CD62L^–^CD8^+^ T cells, NKG2D^+^CD8^+^ T cells, as well as IFN-γ–producing CD8^+^ T cells within indicated populations within the skin after JAK3i treatment. *P < 0.05, ***P < 0.001 (1-way ANOVA). Two replicate experiments were performed for a total of 10 mice per group.

We previously showed that the JAK1/2 inhibitors ruxolitinib and baricitinib, which block both IFN-γ and γc cytokine signaling, showed therapeutic efficacy in the treatment of AA in humans and mice (4, 14). However, the relative contribution of JAK1 versus JAK2 in the pathogenesis of AA is not clear. To define the role of JAK1 and JAK2 in AA, we took advantage of the next generation of JAK1- and JAK2-selective inhibitors to block JAK1 or JAK2 signaling. C3H/HeJ AA mice were treated systemically with INCB039110 (JAK1i), CEP-33779 (JAK2i), or vehicle for 12 weeks. We observed that the mice treated with JAK1i showed robust hair regrowth compared with vehicle-treated mice, which displayed hair loss (Figure 2, A–C). In contrast, CEP-33779 had no effect in restoring hair regrowth and did not prevent further progressive hair loss in all treated mice (Figure 2, A–C). Similar to the effect of CEP-33779 on AA, 2 additional JAK2i (fedratinib and pacritinib) also failed to reverse the disease (Supplemental Table 1, Supplemental Figure 3, and Supplemental Figure 4). Consistent with hair regrowth, immunohistological analysis of skin revealed that JAK1i-treated mice showed substantially reduced histological markers of the disease (CD8, MHC class I, and MHC class II) (Figure 2D). In contrast, the skin of the JAK2i-treated mice displayed massive CD8^+^ infiltrates and increased expression of MHC class I and MHC class II (Figure 2D and Supplemental Figure 3C), similar to vehicle treated.

We next determined the composition of skin infiltrating immune cells after JAKi treatment. We observed a significantly decreased frequency of CD45^+^ immune cells, CD44^+^CD62L^–^CD8^+^ effector/memory T cells (CD8^+^ T_E/M_), and IFN-γ–producing CD8^+^ T cells, in the skin of JAKi1-treated mice, and to a lesser extent, in the skin of JAK2i-treated mice compared with controls (Figure 2, E and F, and Supplemental Figure 3D). Further, treatment with JAK1i significantly reduced the absolute numbers and frequencies of CD8^+^ T_E/M_, CD8^+^NKG2D^+^ T cells, and IFN-γ–producing CD8^+^ T cells within skin draining lymph nodes (SDLNs) compared with controls and JAK2i-treated mice (Supplemental Figure 5). Since JAK1i inhibits both γc cytokine and IFN-γ signaling, and JAK2i inhibits IFN-γ signaling, these data indicate that inhibition of γc cytokine signaling may be sufficient for reversal of AA.

### Systemic treatment with JAK3-selective inhibitor prevented the onset of AA in C3H/HeJ grafted mice.

**Figure 3:**
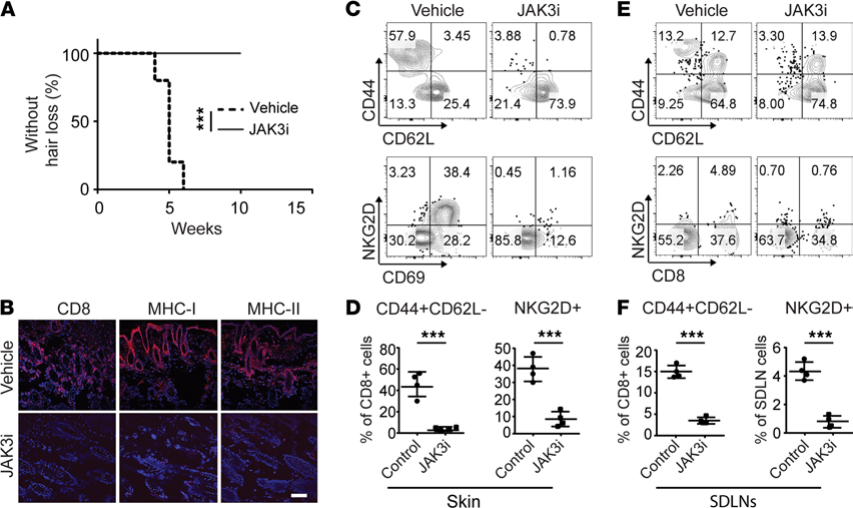
JAK3-selective inhibitor treatment prevented the onset of AA. C3H/HeJ grafted mice were given PF-06651600 (JAK3i) at a dosage of 30 mg/kg for 4 weeks. (**A**) Survival curve analysis depicts the hair loss between JAK3i- and control-treated mice. ***P < 0.01, log-rank test. (**B**) Representative immunofluorescence images of skin sections from JAK3i- or control-treated mice, stained with anti-CD8, anti–MHC class I, or anti–MHC class II mAbs. Scale bar: 200 μm. (**C**) Representative FACS plots of the skin single cell in the viable cell gate were acquired for each sample. (**D**) Summary graphs of the percentages of CD44^+^CD62L^–^CD8^+^ T cells as well as NKG2D^+^CD8^+^ T cells within the skin after treatment. ***P < 0.001 (unpaired Student’s *t* test). (**E**) Representative FACS plots of SDLNs in the viable cell gate were acquired for each sample. (**F**) Summary graphs of the percentages of CD44^+^CD62L^–^CD8^+^ T cells as well as NKG2D^+^CD8^+^ T cells within the SDLNs after treatment. ***P < 0.001 (unpaired Student’s *t* test). Two replicate experiments were performed for 10 mice per group.

We next postulated whether inhibition of γc cytokine signaling alone was sufficient to prevent the onset of AA using JAK3-selective inhibitor. C3H/HeJ skin grafted mice were treated with the JAK3-selective inhibitor PF-06651600 (JAK3i) or vehicle for 4 weeks. The mice were scored for signs of hair loss. As expected, all control mice developed AA by week 7 after skin grafting (Figure 3A). In contrast, the mice that were treated with systemic JAK3i showed no signs of hair loss during the time of observation (Figure 3A). Image analysis of skin revealed that JAK3i-treated mice showed substantially reduced AA-associated skin inflammation, as shown by staining of histological markers of the disease (Figure 3B). Flow cytometric analysis of CD45^+^ subset composition in the skin showed that JAK3i treatment significantly reduced the frequencies of CD8^+^ T_E/M_, as well as NKG2D^+^CD8^+^ T cells, compared with controls (Figure 3, C and D). We previously showed that SDLNs are the primary sites for the development of alopecic T cells (4). Here, we observed that JAK3i significantly reduced the percentages of CD8^+^ T_E/M_ as well as NKG2D^+^CD8^+^ T cells within SDLNs compared with controls (Figure 3, E and F). Taken together, our data demonstrate that the JAK3-selective inhibitor was sufficient to block alopecic T cell proliferation and function and prevented the development of disease.

### Systemic treatment with JAK3-selective inhibitor reversed AA.

**Figure 4:**
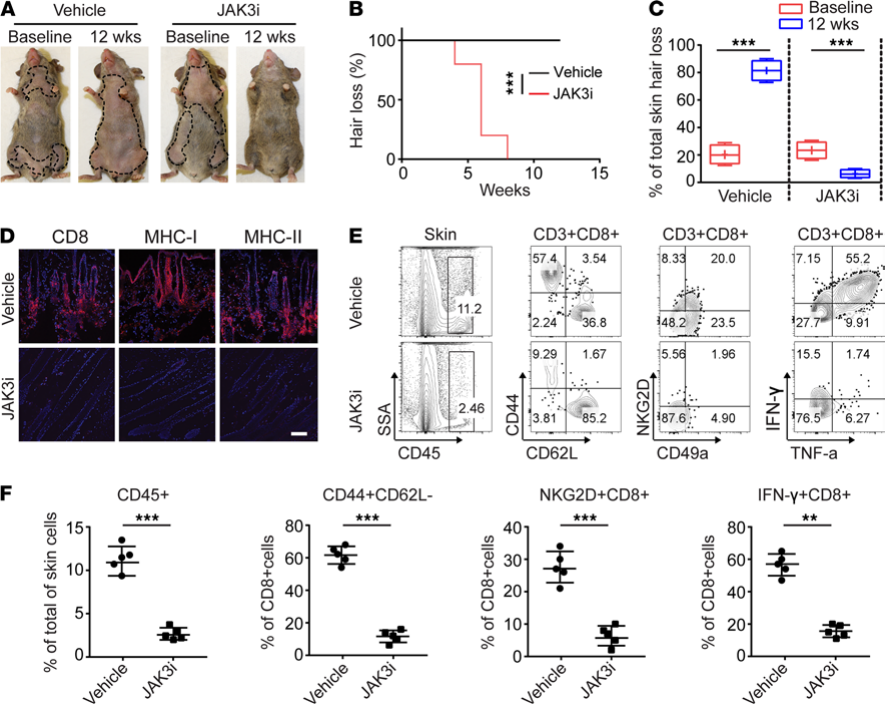
JAK3-selective inhibitor treatment reversed AA. Five C3H/HeJ AA mice per group were treated systemically with PF-06651600 (JAK3i) at a dosage of 30 mg/kg for 12 weeks. (**A**) Representative images of individual JAK3i- or control-treated C3H/HeJ mice before or after 12 weeks’ treatment. (**B**) Time course of hair regrowth shown as weeks after treatment. ***P < 0.001, log-rank test. (**C**) Percentage of total skin hair loss or regrowth shown before and after treatment. The box plots depict the minimum and maximum values (whiskers), the upper and lower quartiles, and the median. The length of the box represents the interquartile range. ***P < 0.001 (unpaired Student’s *t* test). (**D**) Representative immunofluorescence images of skin sections from JAK3i- or vehicle-treated mice, stained with anti-CD8, anti–MHC class I, or anti–MHC class II mAbs. Scale bar: 200 μm. (**E**) and (**F**) Percentages of skin infiltrating CD45^+^ leukocytes, CD44^+^CD62L^–^CD8^+^ T cells, NKG2D^+^CD8^+^ T cells, as well as IFN-γ–producing CD8^+^ T cells within the skin after treatment. **P < 0.01, ***P < 0.001 (unpaired Student’s *t* test). Two replicate experiments were performed for a total of 10 mice per group.

We next assessed the requirement for γc cytokines in AA and the potential of JAK3i to regrow hair in AA mice. After 12 weeks of systemic treatment with PF-06651600 (JAK3i), we observed robust hair regrowth in all treated mice compared with control mice, which showed progressive hair loss (Figure 4, A–C). Consistent with hair regrowth, JAK3i-treated mice showed substantially reduced AA-associated skin inflammation (Figure 4D). Flow cytometric analysis of the CD45^+^ subset composition in the skin revealed that JAK3i-treated mice showed a significantly decreased frequency of CD8^+^ T_E/M_, as well as IFN-γ–producing CD8^+^ T cells, compared with control mice (Figure 4, E and F). Further, treatment with JAK3i significantly reduced the absolute numbers and frequencies of CD8^+^ T_E/M_, CD8^+^NKG2D^+^ T cells, as well as IFN-γ–producing CD8^+^ T cells within SDLNs compared with controls (Supplemental Figure 6, A and B). Taken together, these results underscore the critical role of γc cytokine signaling in established disease. We showed that blockade of γc cytokine signaling by JAK3 inhibition alone suppressed alopecic T cell proliferation and function, and decreased their infiltration into the skin, leading to the reversal of AA.

### Molecular responses of C3H/HeJ AA mice treated with JAK-selective inhibitors.

To define the molecular response to JAKi treatment, we performed RNA-Seq analysis on a series of skin biopsies taken before and after systemic treatment with INCB039110, CEP-33779, PF-06651600, ruxolitinib (JAK1/2i), tofacitinib (pan-JAKi), or vehicle control (Supplemental Table 1). We previously defined biomarkers and molecular responses in patients with AA treated with ruxolitinib or tofacitinib (13, 15). We performed a differential expression analysis between each pre- and posttreatment cohort pair of INCB039110-, CEP-33779–, and PF-06651600–treated skin RNA-Seq samples and performed unsupervised hierarchical clustering on the overlap of the gene lists from all 3 treatments (Supplemental Table 3). The CEP-33779 treatment elicited no significant change in differential expression at appropriate statistical thresholds compared with INCB039110-, PF-06651600–, ruxolitinib-, or tofacitinib-treated mice. Molecular responses were overall consistent across all treatments that resulted in responses (Supplemental Figure 7A). Notably, PF-06651600 treatment elicited the most robust molecular response in terms of both the number of genes modulated (number of inversions in the heatmap), as well as the degree to which they were differentially expressed (overall intensity of color in the heatmap) (Supplemental Figure 7A). Concordantly, Alopecia Areata Disease Activity Index (ALADIN) scores and gene expression profiles correlated with responses with treatment with INCB039110 and PF-06651600, as well as tofacitinib or ruxolitinib (Supplemental Figure 7B).

### Topical JAK1- or JAK3-selective inhibitor treatment restores hair growth in AA mice.

**Figure 5:**
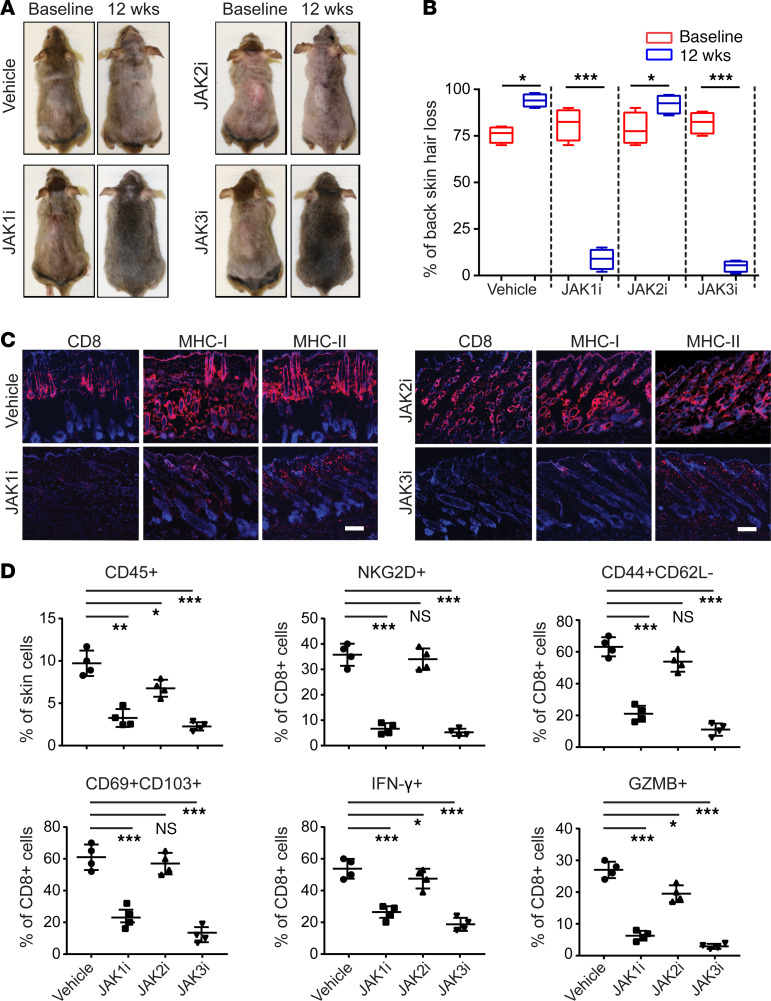
Reversal of AA with topical JAK1- or JAK3-selective inhibitor treatment. C3H/HeJ mice with long standing AA were treated topically with INCB039110 (JAK1i), CEP-33779 (JAK2i), or PF-06651600 (JAK3i) or control daily for 12 weeks, in cohorts of 4 mice per group. (**A**) Representative images of individual JAK inhibitor– or vehicle-treated C3H/HeJ mice before or after 12 weeks’ treatment. (**B**) Percentage of dorsal hair loss or regrowth is shown before and after treatment. The box plots depict the minimum and maximum values (whiskers), the upper and lower quartiles, and the median. The length of the box represents the interquartile range. *P < 0.05, ***P < 0.001 (unpaired Student’s *t* test). (**C**) Representative immunofluorescence images of skin sections from JAKi- or vehicle-treated mice, stained with anti-CD8, anti–MHC class I, or anti–MHC class II mAbs. Scale bar: 100 μm. (**D**) Percentages of skin infiltrating CD45^+^ leukocytes, NKG2D^+^CD8^+^ T cells, CD44^+^CD62L^–^CD8^+^ T cells, CD103^+^CD69^+^CD8^+^ T cells, IFN-γ–producing CD8^+^ T cells, as well as GZMB- or PRF1-producing CD8^+^ T cells within indicated populations within the skin after JAK inhibitor treatment. *P < 0.05, **P < 0.01, ***P < 0.001 (1-way ANOVA). Two replicate experiments were performed for a total of 8 mice per group.

Compared with systemic therapy, topical formulations may offer decreased risk for adverse effects, including serious infections. Topical therapies are an option for treatment of skin inflammatory diseases, including AA, and we previously showed that topical administration of ruxolitinib and tofacitinib effectively reversed AA in C3H/HeJ AA mice (4). We next investigated the effect of topical application of the individual JAK1-, JAK2-, and JAK3-selective inhibitors in AA mice. Consistent with the results of systemic administration, INCB039110 and PF-06651600 robustly restored hair regrowth as early as 6 weeks of daily topical application (Figure 5, A and B). In contrast, treatment with JAK2i (CEP) for as long as 12 weeks had minimal effect on hair regrowth. Immunofluorescence analysis showed that skin from INCB039110- and PF-06651600–treated mice had substantially reduced AA-associated CD8^+^ infiltrates and markers of inflammation compared with JAK2i-treated mice and vehicle controls (Figure 5C). Flow cytometric analysis of skin infiltrating immune cells showed that both INCB039110 and PF-06651600 markedly reduced the frequencies of CD45^+^ infiltrates in the skin, as well as CD8^+^ T effectors (Figure 5D and Supplemental Figure 8). Similarly, topical application of 2 additional JAK2i, fedratinib and pacritinib, showed no significant effect on hair regrowth (Supplemental Figure 9).

To further investigate whether these results were specific to this particular set of JAKi or could be replicated using other JAK-selective inhibitors, we treated additional groups of mice with topical application of an independent set of compounds including GLPG0634 (JAK1i), AZD-1480 (JAK2i), and VX-509 (JAK3i) (Supplemental Table 1). Both GLPG0634 and VX-509 robustly restored hair growth in AA mice, whereas AZD-1480 failed to reverse AA (Supplemental Figure 10), consistent with our findings with the 3 other JAK2i (Figure 5 and Supplemental Figure 9). Our results support the finding that JAK2 inhibition is dispensable for effective treatment of AA.

To exclude the possibility of systemic effects by topical JAKi treatment, we measured the number of CD8^+^ T effector cells in SDLNs. We found that numbers and frequencies of total CD8^+^ T cells, CD8^+^NKG2D^+^ T cells, and CD8^+^ T_E/M_ cells within the SDLNs were not significantly changed by any of the JAK-selective inhibitors compared with control mice (Supplemental Figure 11), suggesting that systemic absorption of topically applied JAK inhibitors was minimal.

## Discussion

This study was designed to interrogate the relative contribution of the γc pathway and IFN-γ pathway in the pathogenesis of AA, using selective JAKi. JAK/STAT signaling plays a critical role in immune system regulation; thus, pharmacological targeting of this pathway has shown promise in the treatment of various immune disorders, including AA (3, 9, 19). Although we previously demonstrated the efficacy of the JAK1/2 inhibitor ruxolitinib and the pan-JAK inhibitor tofacitinib in AA treatment in both C3H/HeJ mice and human patients (4, 13–15), no studies to date have examined the role of selectively inhibiting individual JAKs in the treatment of AA.

Here, we administered a wide range of selective JAKi both systemically and topically in C3H/HeJ AA mice to block individual JAKs and their respective downstream signaling pathways. Our results uncovered a key role of JAK1 and JAK3 signaling in AA, since the inhibition of either pathway by a selective JAK inhibitor was sufficient for disease reversal. Indeed, in vivo exposure to INCB039110, a selective JAK1 inhibitor, or PF-06651600, a selective JAK3 inhibitor, affected a variety of immune processes relevant to both the onset and progression of AA, including the proliferation and activation of alopecic T cells in SDLNs, the secretion of proinflammatory cytokines by skin-infiltrating mononuclear cells, and cytotoxic CD8^+^ T cell–mediated tissue destruction.

The importance of JAK1 and JAK3 signaling in AA pathogenesis is consistent with the central role of γc cytokine signaling in the regulation of lymphocyte development, homeostasis, and function via receptors that contain a unique γc cytokine receptor subunit (8, 9). The γc receptor subunit associates with JAK3 and functions together with JAK1 to phosphorylate and activate STATs for downstream signaling. Previous studies suggested that JAK1 may predominate over JAK3 in the transduction of γc cytokine signaling (20) and that the use of selective JAK3 inhibitors alone might not produce a sufficient immunosuppressive effect to achieve efficacy in the treatment of inflammatory diseases. However, more recent studies showed that JAK1 and JAK3 play equal, albeit nonredundant, roles in propagating γc receptor signaling (22–24). Using a covalent JAK3 inhibitor, it was recently shown that JAK3 is essential for a biphasic pattern of IL-2–induced STAT5 phosphorylation in T cells in vitro (22), raising the possibility that selectively targeting JAK3 may be equally effective as targeting JAK1 for inhibiting γc cytokine signaling (24, 25). Furthermore, the selective JAK3 inhibitor PF-06651600 demonstrated in vivo treatment efficacy in rodent models of adjuvant-induced arthritis and experimental autoimmune encephalomyelitis (21), 2 T cell–mediated inflammatory disorders. In another study, both the covalent JAK3 inhibitor Compound 2, as well as VX-509, a reversible JAK3 inhibitor, achieved partial efficacy in a rat model of collagen-induced arthritis (25, 26), in which T cells are critical to the induction phase of the disease but play a less substantial role in its chronic phase. Taken together, our studies support the hypothesis that inhibiting JAK1 alone or JAK3 alone is sufficient in attenuating γc receptor signaling and reversing AA.

PF-06651600 is an irreversible JAK3-selective covalent inhibitor that potently inhibits JAK3 signaling, but without activity against JAK1, JAK2, and TYK2 (21). This selectivity is achieved via covalent binding to a unique cysteine residue (Cys909) in the catalytic domain of JAK3, which is not present in JAK1, JAK2, or TYK2 (21). Tofacitinib was originally considered a JAK3-selective inhibitor; however, it was later shown that many effects of tofacitinib can be achieved by blockade of JAK3-independent cytokines (27). The efficacy of PF-06651600 in the C3H/HeJ AA mouse model reinforces the notion that γc cytokine signaling inhibition plays a crucial role in reducing disease-related inflammation in AA. Although both JAK1 and JAK3 participate in signaling through γc-containing receptors, unlike JAK1 (which is broadly expressed in many tissues), JAK3 expression is largely restricted to lymphocytes (24, 25).

Both proinflammatory cytokines (such as IFN-γ, IL-6, and γc cytokines) as well as antiinflammatory cytokines (such as IL-10) signal through JAK1. IL-10 has been shown to play a regulatory role in AA (28); however, IL-10–knockout C3H/HeJ mice were relatively resistant to the induction of AA (29). Although the role of IL-10 in AA has yet to be defined, selective JAK3 inhibition would bypass the potential suppression of IL-10 and other antiinflammatory cytokines by JAK1 inhibition, leading to effective disease reversal. Nonetheless, the therapeutic effects of JAK1 inhibition by INCB039110, despite its potential actions on IL-10, suggest that JAK1 and JAK3 may play nonredundant roles in γc receptor signaling and AA pathogenesis. In line with this idea, our gene expression analysis of AA mice before and after treatment with JAKi showed overall consistent responses across all treatment conditions that led to disease reversal, including INCB039110 and PF-00651600.

In our study, treatment with the selective JAK2 inhibitor CEP-33779 failed to reverse disease in C3H/HeJ mice. Furthermore, in our gene expression analyses, AA mouse skin that was treated with CEP-33779 clustered together with pretreatment mice skin and controls, supporting the lack of disease reversal using JAK2 inhibition. To confirm this finding, we then used 3 additional JAK2-selective inhibitors, including fedratinib, pacritinib, and AZD-1480, all of which showed similar results to CEP-33779. Inhibition with JAK2i had no effect on disease reversal in C3H/HeJ mice, further supporting our hypothesis that JAK2 signaling does not play a significant role in AA. JAK2 function is essential for the function of a number of cytokines, including erythropoietin, thrombopoietin, growth hormone, and GM-CSF (30). These cytokines are indispensable for hematopoietic stem cell differentiation and proliferation; thus, potential significant side effects of JAK2 inhibitors may include anemia, thrombocytopenia, and neutropenia. These types of adverse reactions may limit higher dosing of JAKi, since our clinical trials and other studies have shown that high doses of ruxolitinib (JAK1/2 inhibitor) and tofacitinib (pan-JAK inhibitor) were necessary for achieving optimal efficacy in AA (12–15).

IFN-γ signals through JAK1/2 and is a key cytokine implicated in the pathogenesis of AA. We showed in vitro that the JAK1-selective and JAK2-selective inhibitors used in this study equally inhibited IFN-γ–mediated signaling, as expected. However, the 4 JAK2-selective inhibitors in our study had little effect on AA disease reversal. We and others previously showed that IFN-γ plays a critical role in disease induction (31), whereas in chronic AA, numerous inflammatory cytokines (including IFN-γ) have been implicated in the disease (32). Additionally, we and others previously showed that both type I and type II IFN (IFN-γ) signaling pathways are active in the alopecia skin both from human patients with AA and C3H/HeJ mice with AA by gene microarray assays (33). We further confirmed that both type I and type II IFN gene expression signatures were significantly increased with alopecic mouse skin compared with normal-haired C3H/HeJ mice by quantitative PCR assays (data not shown). Type I IFNs signal through JAK1 and TYK2, whereas type II IFNs signal through JAK1 and JAK2, and both type I and type II IFN induce CXCL9/10/11 and STAT1 production (34, 35). Therefore, JAK2 inhibition alone might not inhibit type I IFN response genes such as CXCL9, CXCL11, and STAT1 that can be induced by IFN-γ. However, we cannot formally exclude the possibility that JAK1 compensates in part for JAK2 signaling inhibition in response to IFN-γ (36).

Our previous data showed that CD8^+^ T cells are the main drivers in AA, and these T cells are dependent on γc cytokines for their function and survival (4). Consistent with our results, JAK2i have been shown in other studies to have no significant effect on effector T cell reactivity in a mouse model of graft-versus-host disease, indicating that JAK2 plays little (if any) role in effector T cell function (37). Accordingly, we showed that targeting T cells by inhibiting the γc signaling pathway through JAK1 inhibition or JAK3 inhibition showed therapeutic efficacy in the treatment of AA. Taken together, these findings suggest that the role of IFN-γ in AA pathogenesis is likely to be secondary to γc signaling in the setting of established disease in AA.

Resident memory T cells (T_RM_) persist in peripheral tissues for long periods and play important roles in host defense against infections and tumors. In the skin, pathogenic T_RM_ are involved in a number of inflammatory skin disorders, including psoriasis, vitiligo, and atopic dermatitis (38–42). Alopecic T_RM_ have been reported in patients with AA, but their role in AA remains undefined (43, 44). Most skin infiltrating CD8^+^ T cells in C3H/HeJ AA mice also coexpressed markers characteristic for T_RM_ (CD69^+^CD103^+^). We found that JAK1i or JAK3i treatment significantly decreased the frequency of these T_RM_ in the skin, but not JAK2i treatment. We observed that a small number of T_RM_ remained in the skin of mice even after hair regrowth following JAK1i or JAK3i treatment. Notably, we have observed that many patients with AA began to relapse and lose hair after successful ruxolitinib or tofacitinib treatment (13, 15). One explanation is that a small number of pathogenic T_RM_ may persist in the skin even after successful JAKi treatment, which may become reactivated after stimulation by recovered HF autoantigens upon withdrawal of JAKi treatment, leading to disease relapse. Therefore, a therapeutic strategy that not only inhibits the function of pathogenic T_RM_, but also eliminates them from lesional skin, may produce durable disease remission in AA (39). Future studies will take advantage of the accessibility of the skin by combinations of both topical and systemic JAKi treatment to eradicate the alopecic T_RM_ from lesional skin to achieve prolonged treatment efficacy and durable responses.

In summary, we identified crucial roles of JAK1 and JAK3 signaling in the pathogenesis of AA and showed that blockade of γc signaling alone is sufficient to both reverse and prevent disease. We demonstrated treatment efficacy when the JAKi drugs were administrated either topically or systemically. Our study also defines a mechanistic framework for the use of JAK1-selective or JAK3-selective inhibitors as potent, antiinflammatory agents against AA. These data demonstrate that the selective inhibition of either JAK1 or JAK3 can effectively treat AA, while avoiding the potential adverse effects associated with JAK2 inhibition and without sacrificing treatment efficacy. Unlike JAK1, which is associated with multiple major cytokine receptor families, JAK3 is exclusively associated with the γc cytokine receptor. Therefore, our results indicate that inhibiting JAK3 signaling is sufficient to prevent and reverse disease in the C3H/HeJ model of AA. Our findings invite future clinical trials using novel JAKi that selectively target JAK1 and/or JAK3 for the treatment of AA.

## Methods

### Mice.

C3H/HeJ mice (stock 000659, The Jackson Laboratory) in this study were maintained in a specific pathogen–free environment in a barrier facility, in accordance with the Institutional Animal Care and Use Committee (IACUC) of Columbia University. Transfer of AA was performed using grafted alopecic C3H/HeJ skin or by adoptive T cell transfer as described previously (4).

### JAKi treatment.

JAKi were obtained from different sources: INCB039110 (catalog HY-16997, MedChemExpress), GLPG0634 (filgotinib) (catalog CT-GLPG, ChemieTek), CEP-33779 (catalog 406123, Medkoo), fedratinib (catalog 202893, Medkoo), pacritinib (catalog 202571, Medkoo), AZD-1480 (catalog A-1135, Active Biochem), PF-06651600 (catalog PZ0316, MilliporeSigma), decernotinib (VX-509) (catalog S7541, Selleck), ruxolitinib (catalog S1378, Selleck), and tofacitinib (catalog 200811, Medkoo). For systemic treatment, C3H/HeJ mice with AA were administered INCB039110 (50 mg/kg), CEP-33779 (50 mg/kg), fedratinib (50 mg/kg), pacritinib (50 mg/kg), PF-06651600 (30 mg/kg), ruxolitinib (30 mg/kg), tofacitinib (30 mg/kg), or vehicle control through an ALZET osmotic pump (model 1002, DURECT Corporation). The JAKi were first dissolved in a small volume of DMSO (catalog D12345, Thermo Fisher Scientific) and further were diluted with polyethylene glycol 300 (catalog 202371, MilliporeSigma). For topical treatment, C3H/HeJ AA mice were topically treated with 2% (w/w) various JAKi in Aquaphor (Aquaphor) twice daily, or vehicle control, as we described previously (4). Mice were scored weekly for signs of hair regrowth and loss. Hair loss was monitored and scored according to our previously reported methods (4). Mice were euthanized and organs were collected for analysis after treatment.

### Preparation of tissue cell suspensions.

To prepare skin single-cell suspensions, the skin was cleaned, defatted, and digested in 0.25% trypsin (catalog 15050065, Thermo Fisher Scientific) for 20 minutes at 37°C. Epidermis was separated from the dermis using forceps and scalpel blades. The dermis was finely minced and digested for 45 minutes at 37°C with 2 mg/mL collagenase type IV (catalog CLS-4, Worthington Biochemical Corporation) and 1 μg/mL DNase (catalog DN25, MilliporeSigma) in RPMI 1640 (catalog 61870127, Thermo Fisher Scientific) with 5% FBS (catalog 16000044, Thermo Fisher Scientific) in a shaker. The digested skin was then minced, passed over a 70 μm cell strainer (catalog 229483, CELLTREAT Scientific), and washed before staining. Whole spleen or SDLNs were dissociated and filtered with a 70 μm cell strainer. Splenocytes were depleted of erythrocytes by ACK Lysing Buffer (catalog A1049201, Thermo Fisher Scientific) and washed before staining.

### Antibodies and flow cytometry.

Antibodies used for flow cytometry are listed in Supplemental Table 2. Cells were stained with fixable viability stain LIVE/DEAD Fixable Blue (catalog L23105, Thermo Fisher Scientific) in Dulbecco’s PBS (catalog 14190250, Thermo Fisher Scientific) for 15 minutes at room temperature. Nonspecific antibody binding was blocked using TruStain FcX (catalog 101320, BioLegend). For surface marker staining, cells were incubated with various combinations of fluorochrome-conjugated mAbs in Brilliant Stain Buffer (catalog 563794, BD Biosciences) for 30 minutes at 4°C. The cells were fixed after surface marker staining and then permeabilized using eBioscience Foxp3/Transcription Factor Staining Buffer Set (catalog 00-5523-00, Thermo Fisher Scientific) for detection of intracellular FoxP3, GZMB, PRF1, Ki67, TNF-α, and IFN-γ according to the manufacturer’s instructions. Viable cell populations were gated based on forward and side scatters and by Fixable Blue staining and acquired with an LSRII flow cytometer (BD Biosciences). Analysis was carried out using FlowJo software (Tree Star). Gating strategies for flow cytometry experiments are shown in Supplemental Figure 1.

### In vitro culture and restimulation assays.

For NKG2D^+^CD8^+^ T cell differentiation, splenic T cells were stimulated with 100 ng/mL anti-CD3 in the presence of 10 ng/mL recombinant murine IL-15 (catalog 210-15, PeproTech) and individual JAKi or vehicle control (DMSO) for 72 hours. For intracellular detection of IFN-γ and TNF-α, single-cell suspensions were incubated in 10% FBS RPMI-1640 medium supplemented with Cell Stimulation Cocktail (catalog 00-4970-03, 1:500, Thermo Fisher Scientific) at 37°C. After 1 hour, Brefeldin A (catalog 555029, 1:1000, BD Biosciences) was added, followed by an additional 4-hour incubation at 37°C. The cells were then fixed and permeabilized using the FoxP3 fixation/permeabilization kit and stained intracellularly with anti–IFN-γ and anti–TNF-α for 30 minutes at 4°C.

### STAT phosphorylation assays and Western blot.

Single-cell suspensions of skin or splenocytes were pretreated with the indicated concentration of individual JAKi at 37°C for 60 minutes. The cytokines used were obtained from Peprotech. The treated cells were then incubated with IL-7 (catalog 217-17, 20 ng/mL), IL-10 (catalog 210-10, 50 ng/mL), IL-15 (catalog 210-15, 50 ng/mL), IFN-γ (catalog 315-05, 50 ng/mL), or GM-CSF (catalog 315-03, 50 ng/mL) at 37°C for 15 minutes. Following cytokine treatment, the cells were fixed with 4% paraformaldehyde (catalog AAJ19943K2, Thermo Fisher Scientific) for 15 minutes at room temperature. Fixed cells were then permeabilized with 90% ice-cold methanol (Thermo Fisher Scientific) for 30 minutes on ice and were stained with cell surface markers and anti–p-STAT for 2 hours at room temperature. The isolation of mouse dermal sheath cells was shown previously (4). The dermal sheath cells were pretreated with individual JAKi (0.5 μM final) at 37°C for 60 minutes. The treated cells were then incubated with IFN-γ (50 ng/mL) at 37°C for indicated time points at 37°C. Treated cells were then treated for 20 minutes on ice with RIPA lysis buffer (catalog R0278, MilliporeSigma) supplemented with protease inhibitors (catalog 11697498001, Roche Diagnostics) and phosphatase inhibitor cocktail (catalog P5726, MilliporeSigma). Then 20 μg of total protein was resolved by a 10% SDS-PAGE (catalog 4561033, Bio-Rad) and transferred to PVDF membranes (catalog IPFL85R, MilliporeSigma). After blocking for 60 minutes in 5% nonfat dry milk blocking buffer (catalog sc-2325, Santa Cruz Biotechnology), the membranes were immunoblotted with rabbit anti–p-STAT1 (catalog 7649, 1:1000, Cell Signaling Technology) and rabbit anti-STAT1 (catalog 9172, 1:1000, Cell Signaling Technology), followed by secondary HRP-conjugated anti-rabbit IgG (catalog 7074, 1:1000, Cell Signaling Technology), or anti–β-actin (catalog sc-47778 HRP, 1:5000, Santa Cruz Biotechnology), and chemiluminescent reagent (catalog WBKLS0100, MilliporeSigma). The image was captured and analyzed using ChemiDoc MP Imaging System (Bio-Rad).

### Immunofluorescence.

Inflammatory skin infiltrates and markers were evaluated on frozen skin sections as previously described (4). Acetone-fixed frozen skin sections were blocked in Dulbecco’s PBS + 5% goat serum (catalog S-1000-20, Vector Laboratories) for 1 hour at room temperature. The fixed skin sections were incubated with rat anti-CD8 (catalog 100702, 1:100, BioLegend) and rat anti–I-A/I-E (catalog 107602, 1:100, BioLegend) overnight at 4°C, followed by incubation with Alexa Fluor 594–labeled secondary antibody (catalog A-11007, 1:500, Thermo Fisher Scientific). The endogenous biotin was blocked using a streptavidin/biotin blocking kit (catalog SP-2002, Vector Laboratories). Antifade Mountant with DAPI (catalog H-1200-10, Vector Laboratories) was used as the mounting medium. Immunofluorescence images were captured on a Zeiss LSM 700 laser scanning confocal microscope.

### RNA isolation, RNA-Seq, bioinformatics analysis, and data availability.

Total cellular RNA was extracted using RNeasy Plus Micro Kit (catalog 74034, QIAGEN) from skin homogenates of indicated JAKi-treated mice or controls. RNA quality and quantity were determined using an Agilent BioAnalyzer (Agilent Technologies). Libraries were constructed, pooled, and sequenced on an Illumina HiSeq 4000 at GENEWIZ. The hit counts of each sample were normalized by the DESeq2 package in Bioconductor (45). Differential expression was defined using a significance of FDR less than 0.05 comparing each treated cohort with the vehicle-treated cohort. Unsupervised hierarchical clustering and gene adjacency matrices were generated using Multiple Experiment Viewer. To visualize the difference in expression in the ALADIN genes, the *z* score–normalized hit counts of the ALADIN genes were inputs for clustering (15). Clustering results in this study were done naive to the treatment status of each sample and unblinded at the end. RNA-Seq data sets can be accessed at the National Center for Biotechnology Information’s Gene Expression Omnibus database (https://www.ncbi.nlm.nih.gov/geo/) under the accession code GSE167360.

### Statistics.

Statistical analyses were performed using GraphPad Prism 7.0 software (GraphPad Software Inc.). Groups of data were compared using a 2-tailed Student’s *t* test. Log-rank tests were used to analyze the hair loss or regrowth curves. One-way ANOVA was used for mean differences comparison from multiple groups. All the statistics were conducted using GraphPad Prism software. Data in bar and dot graphs are means ± SEM. Statistically significant *P* values were indicated as follows: *P ≤ 0.05, **P ≤ 0.01, ***P ≤ 0.001, and ****P ≤ 0.0001. Only significant differences (*P* < 0.05) are indicated in the figures. The investigators were not blinded for the analyses.

### Study approval.

All animal experiments were performed in compliance with protocols approved by the IACUC of Columbia University.

## Author contributions

ZD and AMC conceived the study. ZD performed the experiments. JC and YC analyzed data and provided critical review of the manuscript. ZD and AMC analyzed data and wrote the manuscript. AMC supervised the study and provided funding.

## Supplementary Material

Supplemental data

## Version 1 04/08/2021

Electronic publication
