# Supplementary material for: Selective inhibition of JAK3 signaling is sufficient to reverse alopecia areata
Source: JCI Insight. 2021 Apr 8;6(7):e142205. doi: 10.1172/jci.insight.142205 (PMC8119218; doi:10.1172/jci.insight.142205)
Supplement: Supplemental data [file jciinsight-6-142205-s270.pdf]

SUPPLEMENTARY MATERIAL

Selective inhibition of JAK3 signaling is sufficient to reverse alopecia areata

Zhenpeng Dai[1], James Chen[1], Yuqian Chang[1], and Angela M. Christiano[1,2]

Affiliations:
[1] Department of Dermatology, Columbia University, Vagelos College of Physicians & Surgeons, New York, NY, 10032, USA
[2] Department of Genetics & Development, Columbia University, Vagelos College of Physicians & Surgeons, New York, NY, 10032, USA

*Corresponding author Dr. Angela M. Christiano; Email: amc65@cumc.columbia.edu.

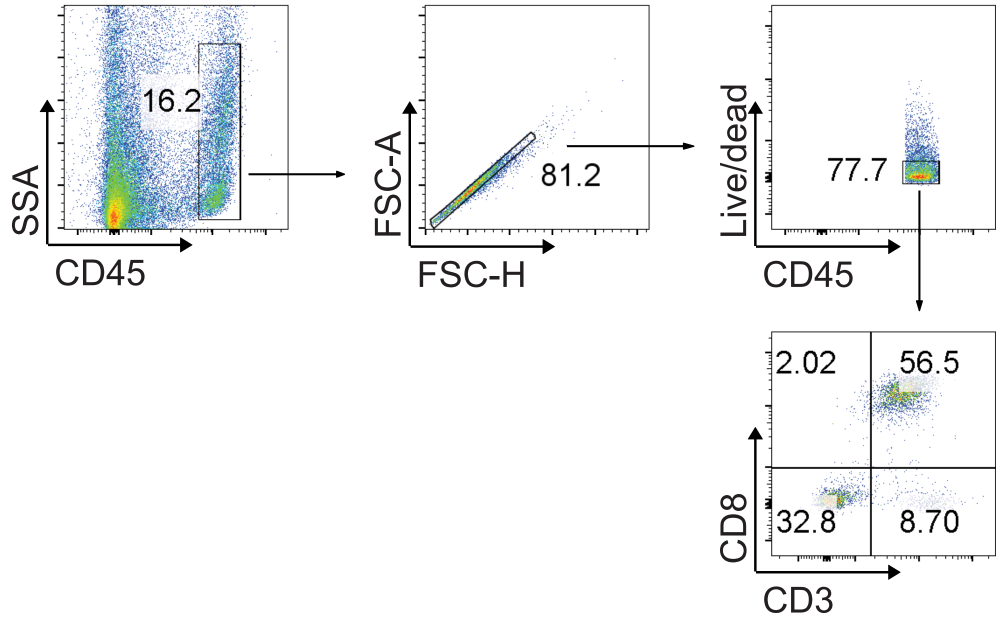

Supplemental Figure 1. Gating strategies for flow cytometric analysis. Single-cell suspensions of skin or lymphoid organs were prepared as described in the Methods. Leukocytes within the skin or lymphoid organs were gated as $CD45^{+}$. Viable cell populations were gated based on forward and side scatters and by Fixable Blue staining. T cells were gated as $CD45^{+}CD3^{+}$ and were further analyzed for the expression of indicated markers.

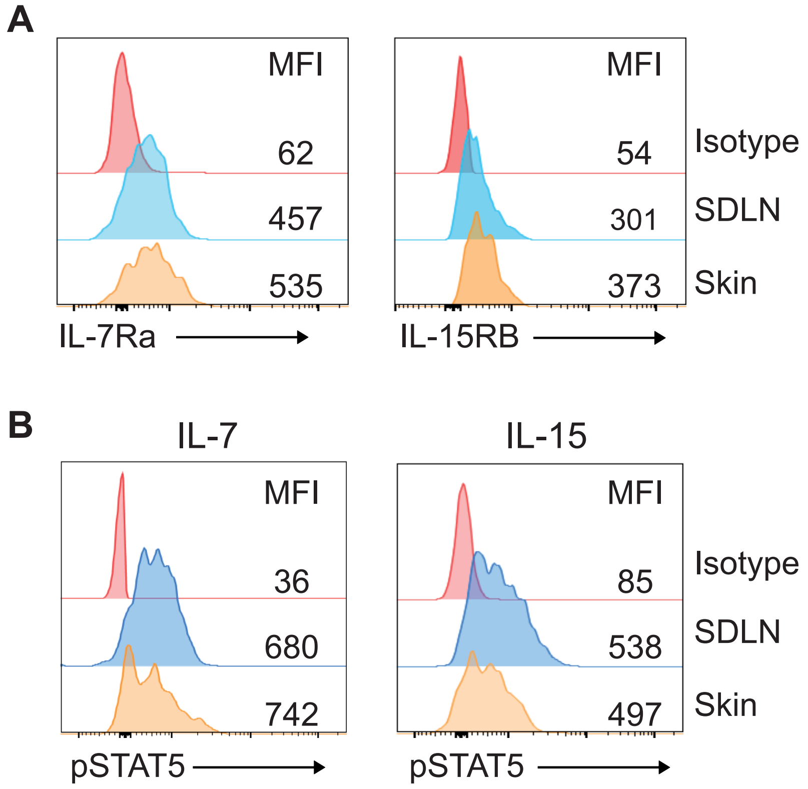

Supplemental Figure 2. AA skin infiltrating CD8⁺ T cells were responsive to γc cytokine stimulation. Expression of γc cytokine receptors in AA lesional infiltrating T cells. (**A**) Flow cytometric analysis of IL-7Ra and IL-15RB expression by CD8⁺ T cells within SDLN and skin from C3H/HeJ AA mice, presented as representative plots and mean fluorescence intensity (MFI). (**B**) Flow cytometric analysis of pSTAT5 expression in CD8⁺ T cells within indicated tissues after treatment for 20 min with IL-7 (20 ng/mL) or IL-15 (20 ng/mL), presented as representative plots and mean fluorescence intensity (MFI) for pSTAT5 expression.

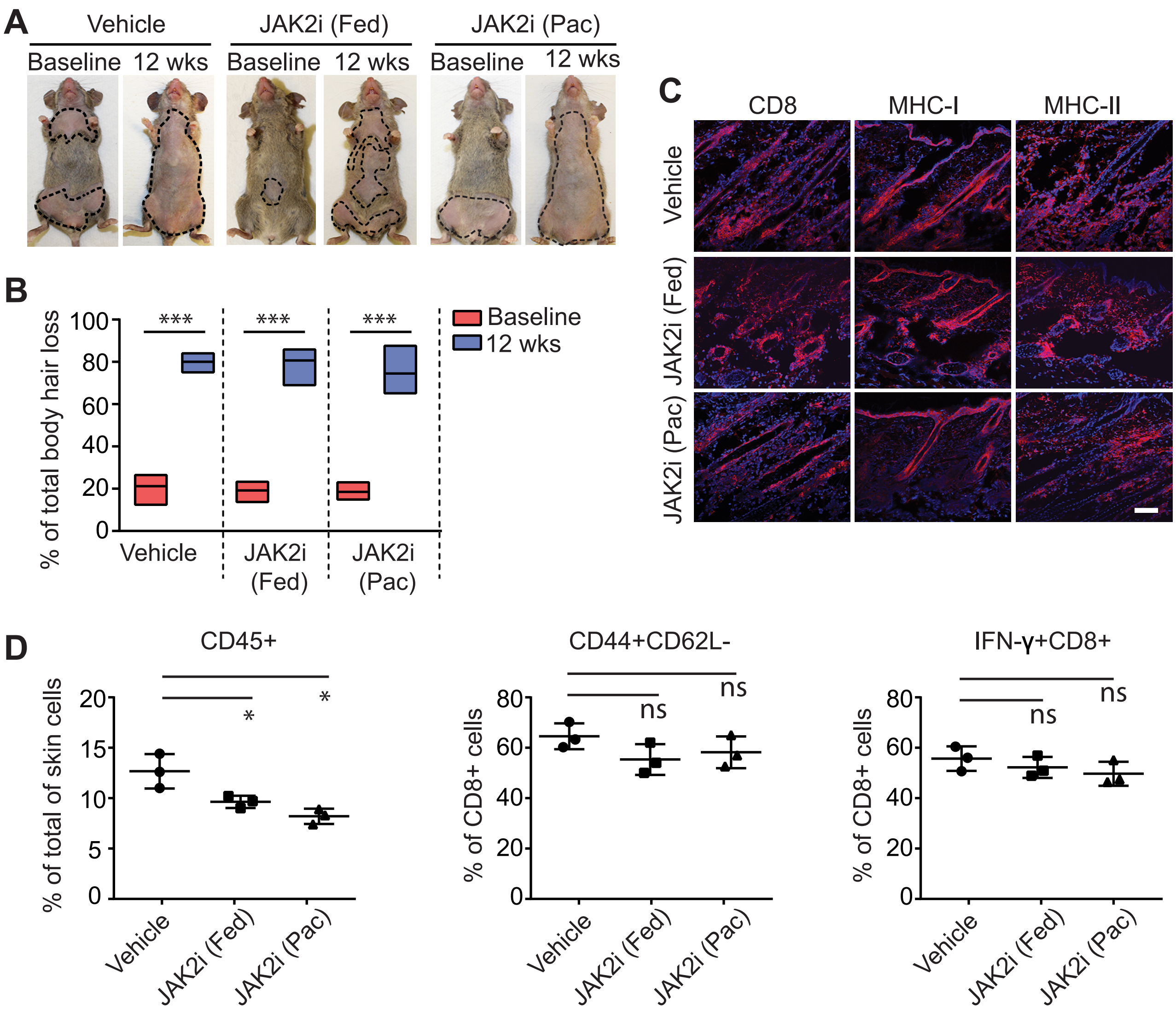

Supplemental Figure 3. JAK2-selective inhibitor treatment failed to reverse AA, Related to Fig. 2. Mice were treated as in Fig.2. Three C3H/HeJ AA mice per group were given Fedratinib (JAK2i (Fed)), Pacritinib (JAK2i (Pac)) or Vehicle at a dosage of 50mg/kg for 12 wks. (**A**) Representative images of individual JAK inhibitors or vehicle control-treated C3H mice before or after 12 weeks treatment. (**B**) Percentage of skin loss or regrowth were shown before and after treatment. ***P< 0.001(Unpaired Student's t-test). (**C**) Representative immunofluorescence images of skin sections from JAK inhibitors or vehicle control-treated mice, stained with anti-CD8, anti-MHC-I, or anti-MHC-II mAbs. Dashed scale bars represent 100 μm. (**D**) Percentages of skin infiltrating $CD45^+$ leukocytes, $CD44^+CD62L^-CD8^+$ T cells, as well as IFN-γ producing $CD8^+$ T cells within the skin after treatment. ns indicates not significant, *P < 0.05, ***P < 0.001 (one-way ANOVA). Two replicate experiments were performed for a total 6 mice per group.

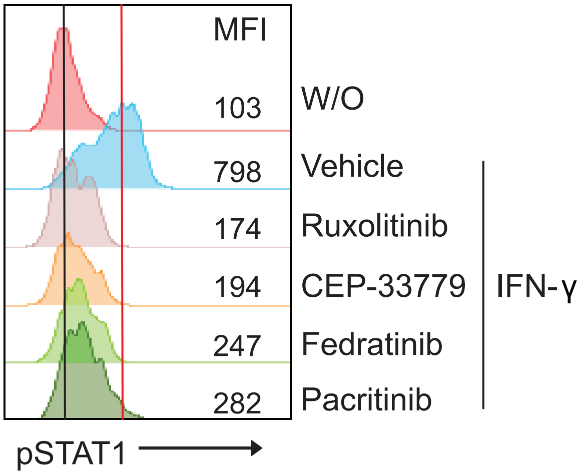

Supplemental Figure 4. JAK2 selective inhibitors inhibit cytokine-induced JAK2 signaling in vivo, Related to Figure 2 and Supplementary Figure 3. JAK2 selective inhibitors inhibit cytokine-induced JAK2 signaling *in vivo*. C3H/HeJ mice were administered by CEP-33779 (50 mg/kg), Fedratinib (50 mg/kg), Pacritinib (50 mg/kg), Ruxolitinib (30 mg/kg), or vehicle control through an ALZET osmotic pump for 7d. Peripheral blood was collected, stimulated with 50 ng/ml IFN-γ, and analyzed by flow cytometry for pSTAT1.

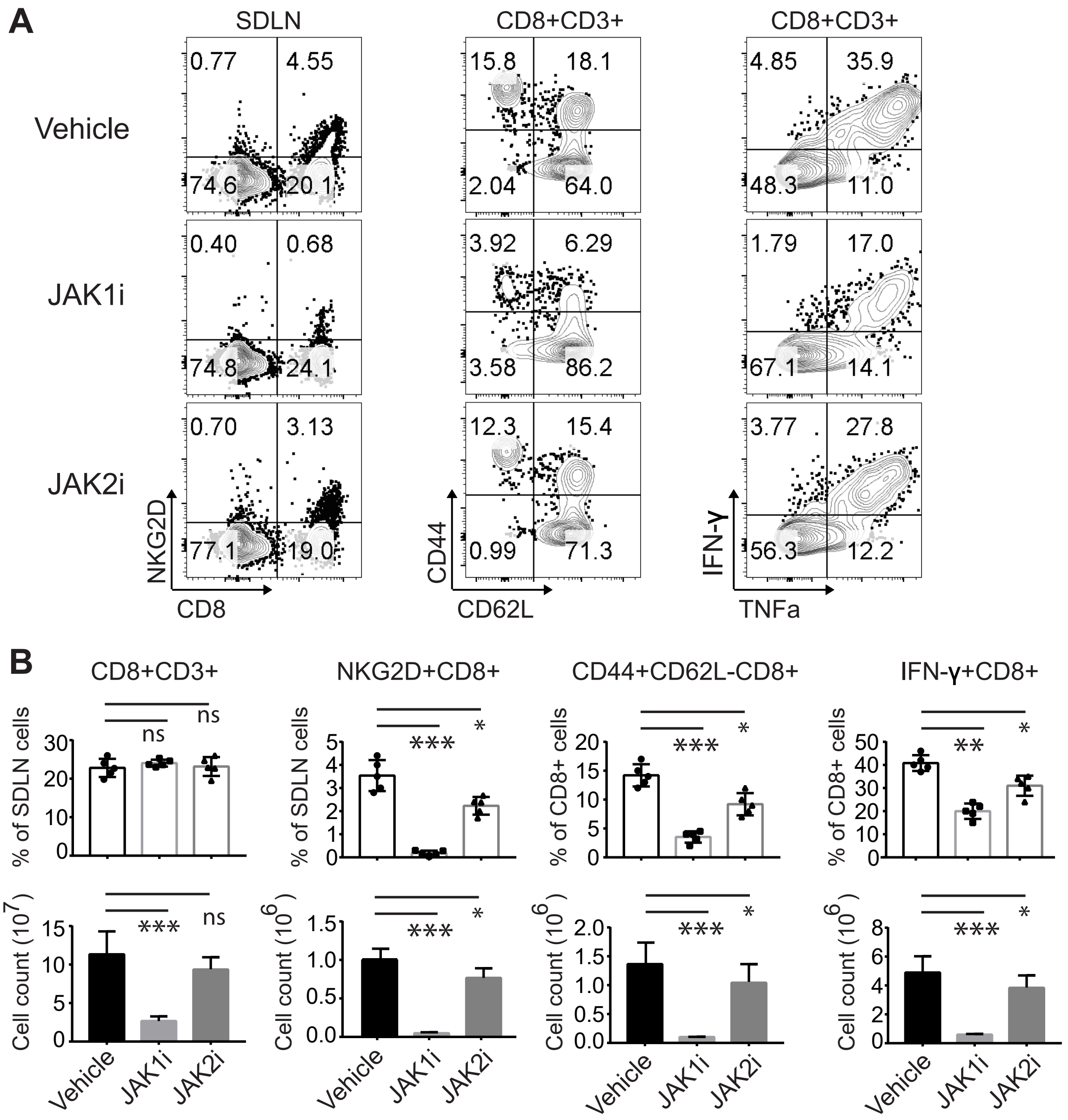

Supplemental Figure 5. The effect of JAK1 or JAK2 blockade in peripheral lymphoid organs, Related to Figure 2. Mice were treated as in Fig.4. (**A**) Representative flow cytometric plots analysis of expression of NKG2D, CD44 or CD62L on $CD8^+$ T cells and IFN-γ producing $CD8^+$ T cells. (**B**) Summary graphs of the percentages and total numbers of $CD8^+$ T cells, $CD44^+CD62L^-CD8^+$ T cells, $NKG2D^+CD8^+$ T cells, as well as IFN-γ producing $CD8^+$ T cells within SDLN after treatment. n.s. indicates not significant, *P < 0.05, **P < 0.01, ***P < 0.001(one-way ANOVA). Two replicate experiments were performed for a total 10 mice per group.

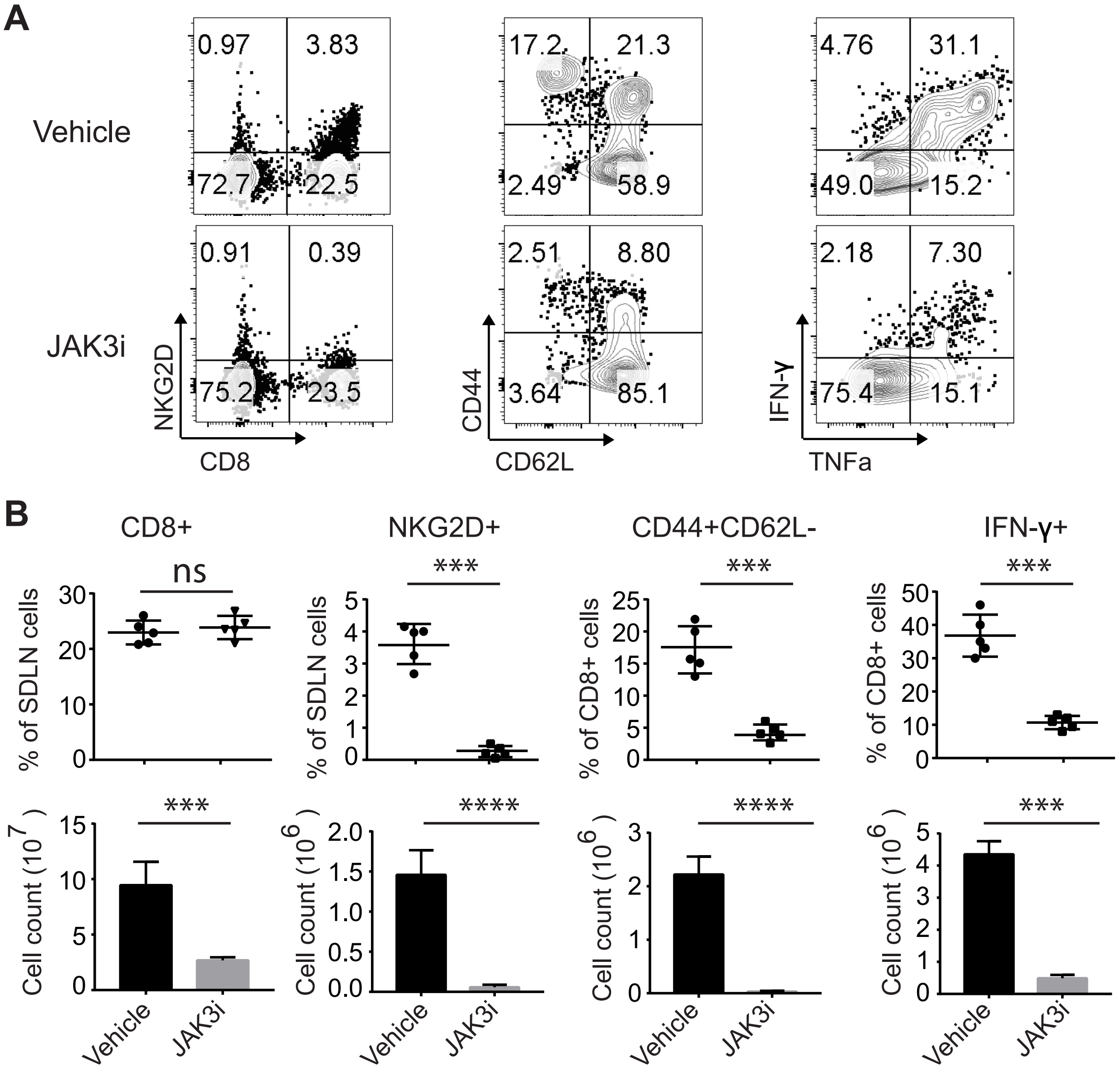

Supplemental Figure 6. The effect of JAK3 blockade in peripheral lymphoid organs, Related to Figure 4. Mice were treated as in Fig.3. (**A**) Representative flow cytometric plots analysis of expression of NKG2D, CD44 or CD62L on $CD8^+$ T cells and IFN-γ producing $CD8^+$ T cells. (**B**) Summary graphs of the percentages and total numbers of $CD8^+$ T cells, $CD44^+CD62L^-$ $CD8^+$ T cells, $NKG2D^+CD8^+$ T cells, as well as IFN-γ producing $CD8^+$ T cells within SDLN after treatment. n.s. indicates not significant, ***P < 0.001 (Unpaired Student's t-test). Two replicate experiments were performed for a total 10 mice per group.

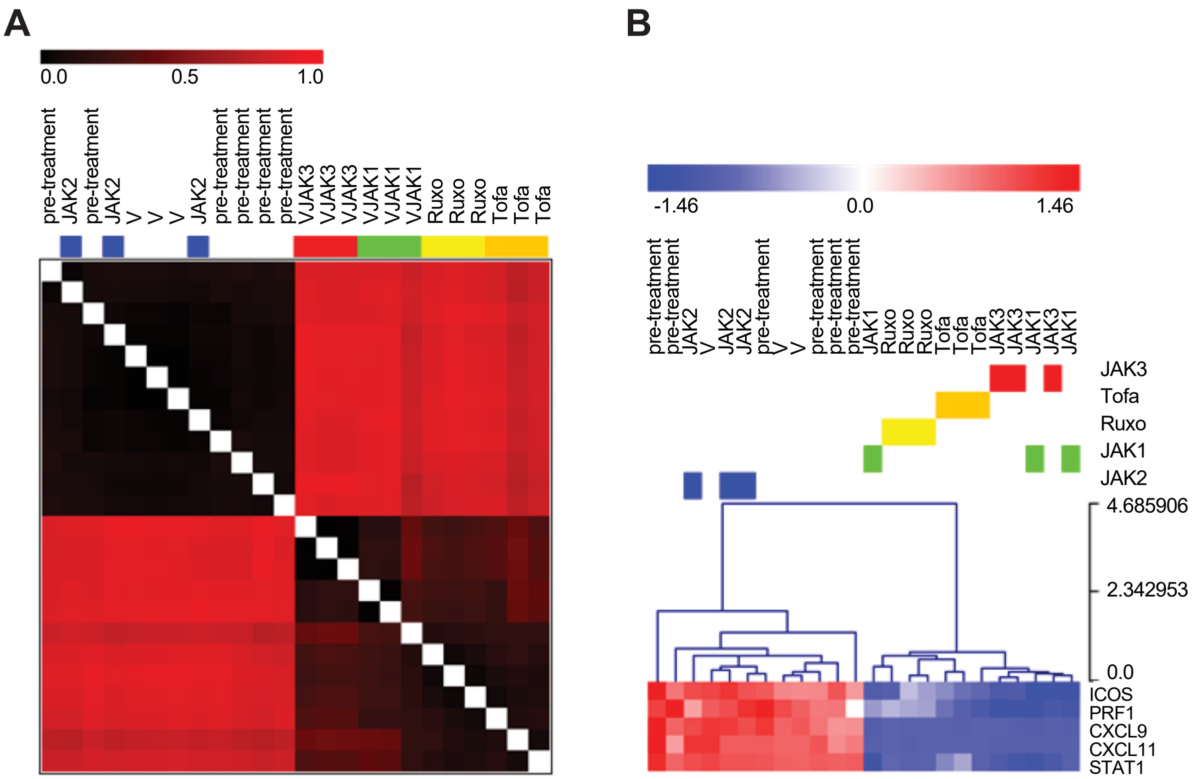

Supplemental Figure 7. Molecular analysis of skin biopsies taken from JAKi treated mice. Related to Figure 2 and Figure 4. Mice were treated as described in Fig.2 and Fig.4. (A) Clustering results of RNA-seq analysis expressed as an adjacency matrix. Stronger red indicates molecular similarity between sample pairs (row against column). (B) ALADIN CTL signature genes were investigated using RNA-seq, including ICOS, PRF1, CXCL9, CXCL11, and STAT1. The heat map indicates that JAK1i, JAK3i, ruxolitinib and tofacitinib (blue shaded area) suppressed the ALADIN CTL signature, compared to JAK2i, and vehicle and pretreatment controls, where expression remained high (red shaded area). Abbreviation: V, Vehicle; JAK1, INCB039110; JAK2, CEP-33779; JAK3, PF-06651600; Ruxo, ruxolitinib; Tofa, tofacitinib.

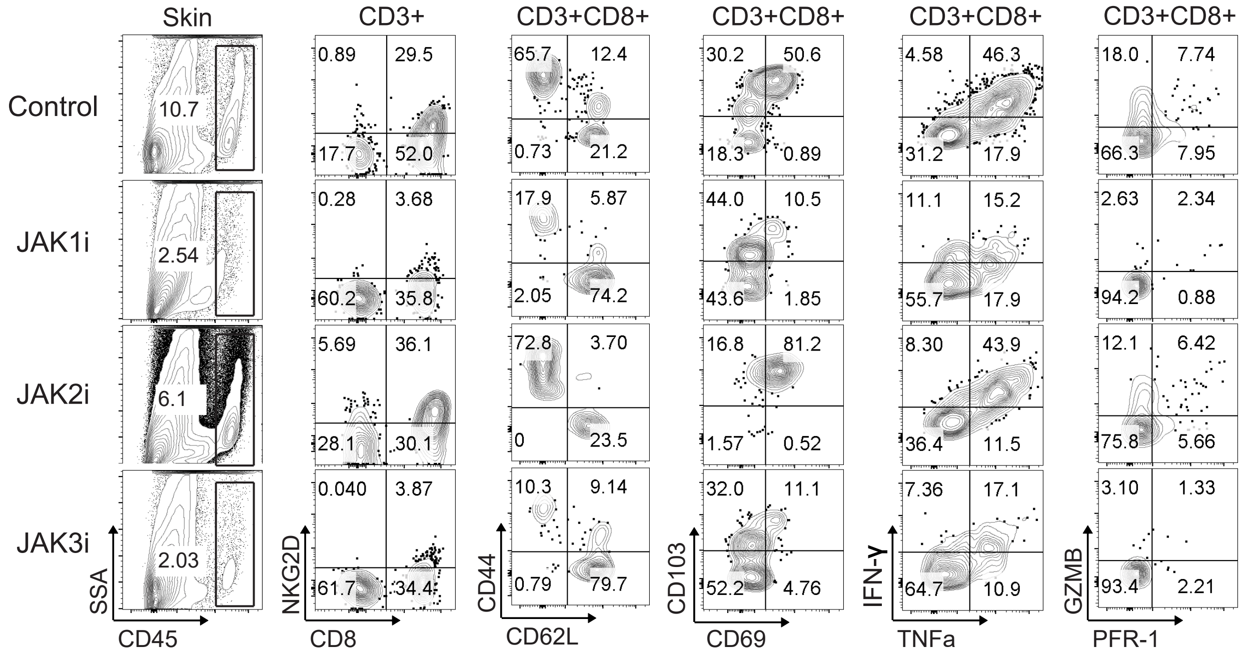

Supplemental Figure 8. Topical JAK-selective inhibitor treatment alters T cell activation in skin, Related to Figure 6. Mice were treated as in Fig. 6. Representative flow cytometric plots showing the frequencies of CD45$^+$ leukocytes, CD8$^+$ T cells, CD103$^+$CD69$^+$CD8$^+$ T cells, CD44$^+$CD62L$^-$CD8$^+$ T cells, IFN-γ producing CD8$^+$ T cells, GZMB and PRF1 producing CD8$^+$ T within the skin after JAK inhibitors treatment.

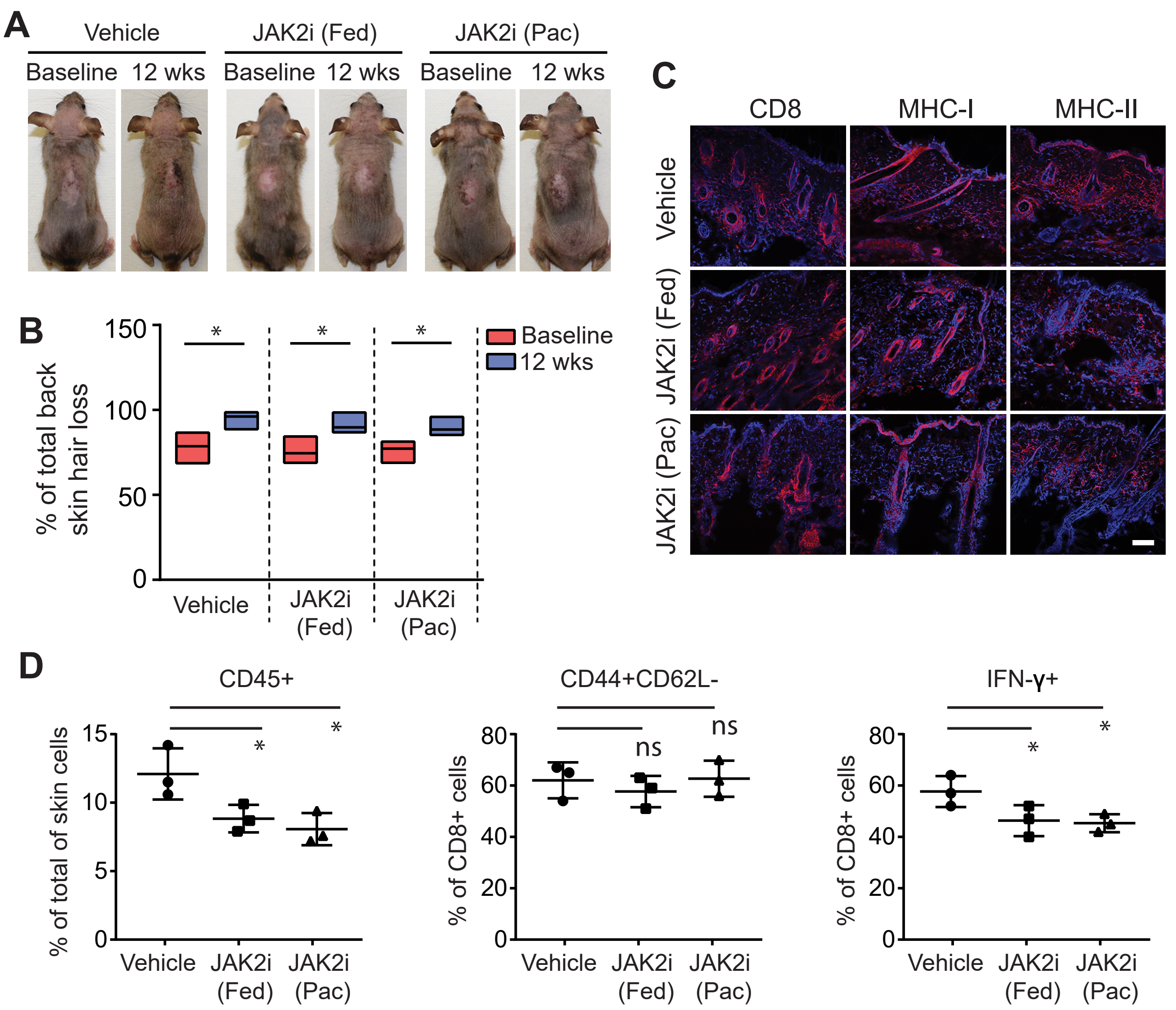

Supplemental Figure 9. Topical JAK2-selective inhibitor treatment failed to induce hair growth in C3H/HeJ mice with AA, Related to Fig. 6. Mice were treated as in Fig. 6. Three long standing C3H/HeJ AA mice per group were topically applied with Fedratinib (JAK2i), Pacritinib (JAK2i) or vehicle control daily for 12 wks. (**A**) Representative images of individual JAK inhibitors or vehicle control-treated C3H/HeJ mice before or after 12 weeks treatment. (**B**) Percentage of skin dorsal skin hair loss or regrowth were shown before and after treatment. *P< 0.05 (Unpaired Student's t-test). (**C**) Representative immunofluorescence images of skin sections from JAK inhibitors or vehicle control-treated mice, stained with anti-CD8, anti-MHC-I, or anti-MHC-II mAbs. Dashed scale bars represent 100 μm. (**D**) Percentages of skin infiltrating $CD45^+$ leukocytes, $CD44^+CD62L^-CD8^+$ T cells, as well as IFN-γ producing $CD8^+$ T cells within the skin after JAK inhibitors treatment. ns indicates not significant, *P < 0.05 (one-way ANOVA). Two replicate experiments were performed for a total 6 mice per group.

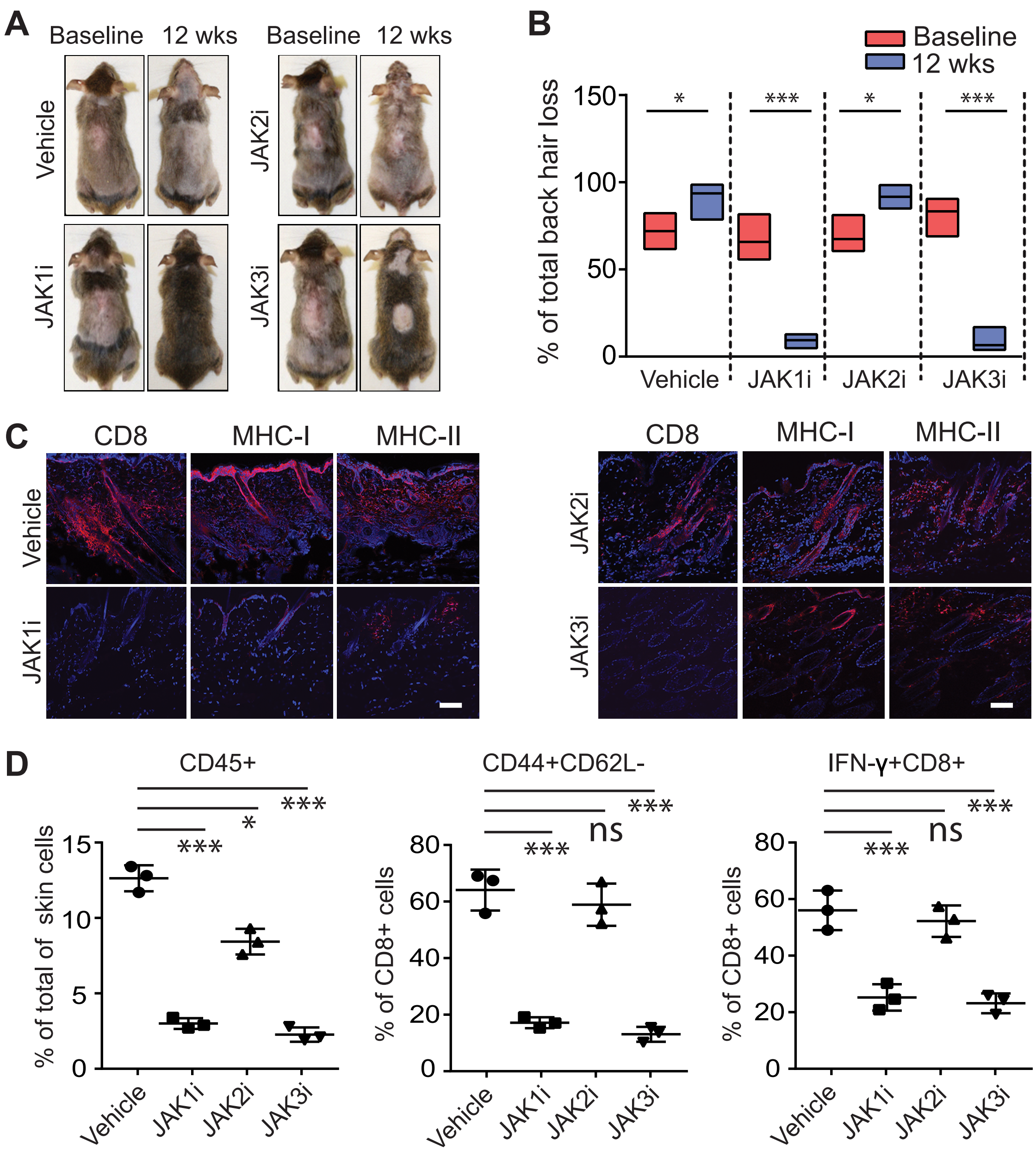

Supplemental Figure 10. Topical JAK1-selective or JAK3-selective inhibitor treatment induced hair growth in C3H/HeJ mice with AA, Related to Fig. 6. Mice were treated as in Fig. 6. Three long standing C3H/HeJ AA mice per group were topically applied with GLPG0634 (JAK1i), AZD1480 (JAK2i), and VX-509 (JAK3i) or vehicle control daily for 12 wks. (**A**) Representative images of individual JAK inhibitors or vehicle control-treated C3H mice before or after 12 weeks treatment. (**B**) Percentage of dorsal skin hair loss or regrowth were shown before and after treatment. *P < 0.05, ***P < 0.001(Unpaired Student's t-test). (**C**) Representative immunofluorescence images of skin sections from JAK inhibitors or vehicle control-treated mice, stained with anti-CD8, anti-MHC-I, or anti-MHC-II mAbs. Dashed scale bars represent 100 μm. (**D**) Percentages of skin infiltrating $CD45^+$ leukocytes, $CD44^+CD62L^-CD8^+$ T cells, as well as IFN-γ producing $CD8^+$ T cells within the skin after JAK inhibitors treatment. ns indicates not significant, *P < 0.05, ***P < 0.001(one-way ANOVA). Two replicate experiments were performed for a total 6 mice per group.

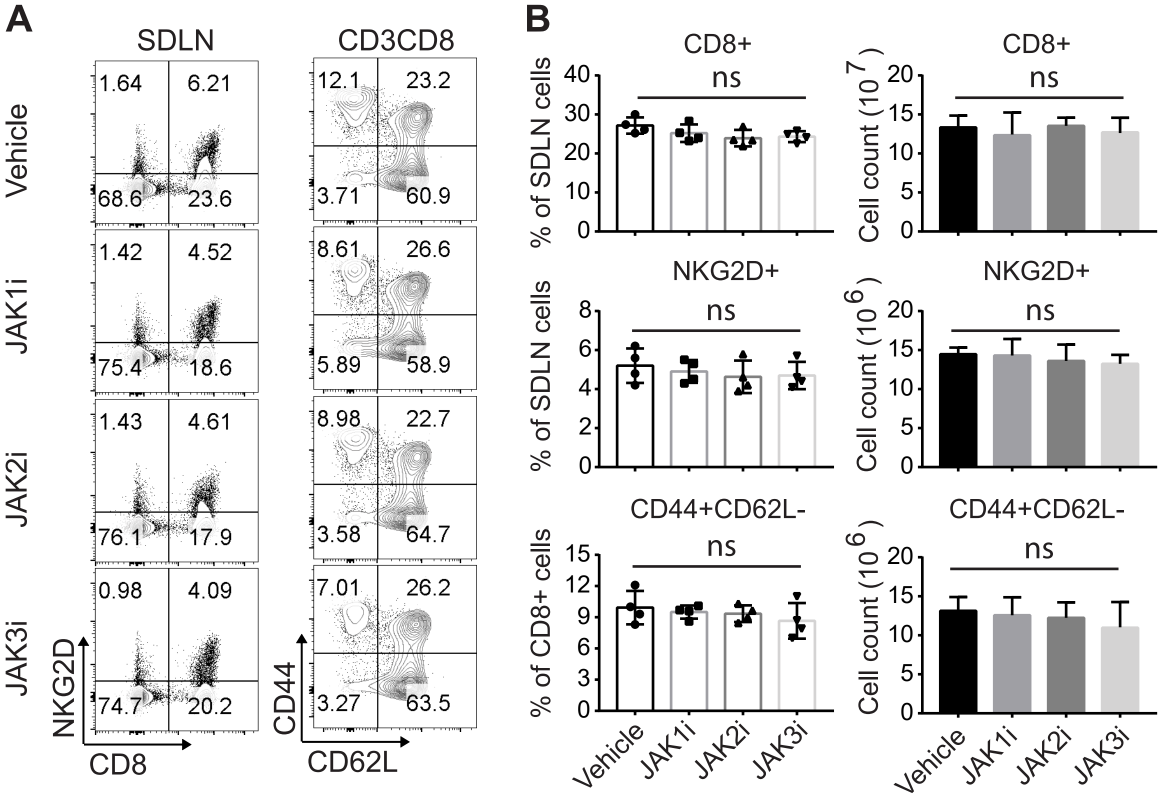

Supplemental Figure 11. Topical JAK-selective inhibitor treatment had no significant effect on T cell activation in peripheral lymphoid organs, Related to Fig. 6. Mice were treated as in Fig. 6. (**A**) Representative flow cytometric plots showing the expression of NKG2D, CD44 and CD62L on $CD8^+$ T cells within SDLNs after treatment. (**B**) Summary graphs of the percentages and total numbers of $CD8^+$ T cells, $CD44^+CD62L^-CD8^+$ T cells ae well as $NKG2D^+CD8^+$ T cells within the SDLN after JAK inhibitors treatment. ns indicates not significant (one-way ANOVA).

Supplemental Table 1. Kinase selectivity profile of the JAK inhibitors used in this study in cell-free assays.

Target / Drug	JAK1 IC50 (nM))	JAK2 IC50 (nM))	JAK3 IC50 (nM))	TYK2 IC50 (nM))
INCB03911 (1)	2	63	2000	795
Filgotinib (2)	10	28	810	116
CEP33779 (3)	72	1.8	85	1400
Fedratinib (4)	105	3	1002	405
Pacritinib (5)	1280	23	520	50
AZD1480 (6)	1.3	< 0.4	3.9	N/D
PF-06651600 (7)	>10000	>10000	33.1	>10000
Decernotinib (8)	11	13	2.5	11
Ruxolitinib (9)	3.3	2.8	428	19
Tofacitinib (10)	15.1	77.4	55.0	489.0

References

1. Kettle JG, Astrand A, Catley M, Grimster NP, Nilsson M, Su Q, et al. Inhibitors of JAK-family kinases: an update on the patent literature 2013-2015, part 2. *Expert Opin Ther Pat.* 2017;27(2):145-61.
2. Van Rompaey L, Galien R, van der Aar EM, Clement-Lacroix P, Nelles L, Smets B, et al. Preclinical characterization of GLPG0634, a selective inhibitor of JAK1, for the treatment of inflammatory diseases. *J Immunol.* 2013;191(7):3568-77.
3. Stump KL, Lu LD, Dobrzanski P, Serdikoff C, Gingrich DE, Dugan BJ, et al. A highly selective, orally active inhibitor of Janus kinase 2, CEP-33779, ablates disease in two mouse models of rheumatoid arthritis. *Arthrit Res Ther.* 2011;13(2):R68.
4. Wernig G, Kharas MG, Okabe R, Moore SA, Leeman DS, Cullen DE, et al. Efficacy of TG101348, a selective JAK2 inhibitor, in treatment of a murine model of JAK2V617F-induced polycythemia vera. *Cancer Cell*. 2008;13(4):311-20.
5. Hart S, Goh KC, Novotny-Diermayr V, Hu CY, Hentze H, Tan YC, et al. SB1518, a novel macrocyclic pyrimidine-based JAK2 inhibitor for the treatment of myeloid and lymphoid malignancies. *Leukemia.* 2011;25(11):1751-9.
6. Hedvat M, Huszar D, Herrmann A, Gozgit JM, Schroeder A, Sheehy A, et al. The JAK2 inhibitor AZD1480 potently blocks Stat3 signaling and oncogenesis in solid tumors. *Cancer Cell*. 2009;16(6):487-97.
7. Telliez JB, Dowty ME, Wang L, Jussif J, Lin T, Li L, et al. Discovery of a JAK3-Selective Inhibitor: Functional Differentiation of JAK3-Selective Inhibition over pan-JAK or JAK1-Selective Inhibition. *ACS Chem Biol.* 2016;11(12):3442-51.
8. Mahajan S, Hogan JK, Shlyakhter D, Oh L, Salituro FG, Farmer L, et al. VX-509 (decernotinib) is a potent and selective janus kinase 3 inhibitor that attenuates inflammation in animal models of autoimmune disease. *J Pharmacol Exp Ther*. 2015;353(2):405-14.
9. Quintas-Cardama A, Vaddi K, Liu P, Manshouri T, Li J, Scherle PA, et al. Preclinical characterization of the selective JAK1/2 inhibitor INCB018424: therapeutic implications for the treatment of myeloproliferative neoplasms. *Blood.* 2010;115(15):3109-17.
10. Flanagan ME, Blumenkopf TA, Brissette WH, Brown MF, Casavant JM, Shang-Poa C, et al. Discovery of CP-690,550: a potent and selective Janus kinase (JAK) inhibitor for the treatment of autoimmune diseases and organ transplant rejection. *J Med Chem.* 2010;53(24):8468-84.

Supplemental Table 2. Antibodies used for Flow Cytometry.

Antibody-Conjugate	Company (clone)	Catalog Number	Dilution
CD3-PerCP-eFluor 710	Thermo Fisher Scientific (17A2)	46-0032-82	1:100
CD4-BUV496	BD Bioscience (GK1.5)	612952	1:100
CD4-BV711	BD Bioscience (GK1.5)	563050	1:200
CD8-BUV395	BD Bioscience (53-6.7)	563786	1:200
CD8-PE/Cyanine7	Biolegend (53-6.7)	100722	1:200
CD11b-APC	Biolegend (M1/70)	101212	1:200
CD19-PE/Dazzle 594	Biolegend (6D5)	115554	1:200
CD25-BV650	Biolegend (PC61)	102038	1:200
CD44-FITC	BD Bioscience (IM7)	553133	1:100
CD45-Alexa Fluor 700	Biolegend (30-F11)	103128	1:200
CD45-APC/Cyanine7	Biolegend (30-F11)	103116	1:200
CD49a-Alexa Fluor 647	BD Bioscience (Ha31/8)	562113	1:200
CD62L-APC-R700	BD Bioscience (MEL-14)	565159	1:200
CD69-BV785	Biolegend (H1.2F3)	104543	1:200
CD103-BV421	Biolegend (2E7)	121422	1:200
CD122-APC	Biolegend (TM-β1)	123214	1:200
CD127-BV421	Biolegend (A7R34)	135027	1:200
NKG2D-PE	Biolegend (CX5)	130208	1:100
GZMB-FITC	Biolegend (GB11)	515403	1:50
PRF1-APC	Biolegend (S16009A)	154304	1:200
Ki67-eFluor 450	Thermo Fisher Scientific (SolA15)	48-5698-82	1:100
FoxP3-PE-eFluor 610	Thermo Fisher Scientific (FJK-16s)	61-5773-82	1:200
IFN-γ-FITC	Biolegend (XMG1.2)	505806	1:100
TNF-α-PE/Cyanine7	Biolegend (MP6-XT22)	506324	1:200
Phospho-Stat1-PE	Cell Signaling Technology (D4A7)	25809S	1:50
Phospho-Stat3-PE	Cell Signaling Technology (D3A7)	8119S	1:50
Phospho-Stat5-PE	Cell Signaling Technology (C71E5)	5387S	1:50

Supplemental Table 3

jak1	jak2	jak3
Slc2a9		0610012G03Rik
Cd69		Zcchc2
Ifit1		Bcat2
Rprd1b		A930005H10Rik
Oas1g		Sdr42e1
Rnf213		Egr1
Ifi44		1500011K16Rik
Gm10648		Mctp2
Ogfr		Myo5b
Gm4951		Cxadr
Dhrs9		Slc10a6
Ceacam12		Rarres2
Mlkl		Atp5c1
Slfn9		Prdm1
Iars2		Sorbs3
Parp9		Irgm1
Parp14		Kmt5a
Pinlyp		Trim56
Pmepa1		Arl5a
Slc39a2		Map2k4
Aasdh		Erp44
Erap1		Ap1s3
Xaf1		Tsc22d2
Oas1a		Adam10
Slc25a13		Commd3
Plekhb1		Cldn5
Dnajb4		Chchd3
Ubxn11		Adgrf2
Aebp2		Cdsn
Il4ra		Tmprss4
Creld1		Rps6ka5
Cd55		Dmkn
Ccdc88b		Tmtc3
AW112010		Itpkb
Stat2		Stat3

Trim21	Rcbtb1
Smox	Sorcs2
Slfn2	Ifih1
Tnfrsf14	Ivl
Cxcl16	Carmil1
Pitpnm1	Ap1m2
Pccb	Ttc22
Oasl2	Map3k9
Zfp383	Runx3
Ehf	Prf1
Igsf3	Ppfia3
Coro2a	Spry2
Wnt11	Rassf10
Oas2	Luzp1
Gbp3	Zfp763
Ccdc62	Mical2
Padi4	Cygb
Asb2	Bvht
H2-T24	Cpeb1
Ifih1	Ubxn11
Pqlc1	Arl15
Aif1	Srpk1
Kansl1l	Dsp
Teddm3	Lipm
Carmn	Fut1
Krtap4-9	Adar
Ddx24	Mfhas1
C4a	Igsf9
Cdh13	Blzf1
Phf11b	Phip
B4galnt1	Fhl2
Efhd2	Sesn2
Porcn	Ptprf
Adam1a	Cdc42ep3
Car9	Gpsm1
Capg	Lsr
H2-Q1	Ndufs6
Samd5	Upf3b
H2-T22	Rab27b
Psme1	Zfp606
Helz2	Psmb10

Itgb2	Grhl1
Styk1	Ppfia1
St6galnac2	Uqcc1
Uba7	Ndufa13
H2-Bl	Sh3tc1
Rtp4	Esrp1
Il2rb	Plcxd2
Eno1	Parp9
Fnip1	Trim59
Trappc9	Jup
Cdc42ep3	Ankrd22
Serpinb1a	Cyp4f39
2-Mar	Cdcp1
Casp3	Crtc2
Trim34b	Serpinb6a
Mtus1	Sec24c
Lgals4	Larp4b
Ndufa4l2	Arhgef5
BC067074	Rassf5
Mill1	Tinf2
Acp6	Aak1
Tmem268	Trip12
Pfkl	Ndufa1
Mir142	Acpp
Scmh1	Pdlim2
Kdm5a	Dlg3
Slc2a12	Pla2g4f
Gpaa1	Fcho1
Csf3r	Hip1r
Slc2a1	Ndufb11
Nlrc5	Daam1
H2-K2	Eml4
Stil	Klf3
Plbd1	Ndufb5
Flt1	Tuft1
Piezo1	Kat6a
	Ak4
	Fam83h
	Esrp2
	Celsr2
	Mill1

Dedd2
Ptk6
Gpr87
Carmn
Afap1l2
Kdm5c
Aldh3b3
Unc119
Plxnb2
Sec31a
Zfp266
Tango2
Arhgef37
Gtpbp3
Grb7
Defb1
Zdhhc9
Zfp169
Hint2
Cldnd1
Mtfr1
Ndufa5
Rangap1
Baz2a
Npepl1
Csnk1a1
Klhl25
Pof1b
Arih1
Arhgap18
Fmc1
Usp34
Ano7
Unc45a
Slc9a8
Sbno1
Sptbn2
Plppr3
Gale
Kdm5b
Ddrgk1

Ceacam1
Pkn1
Ocln
Serpinb12
Pex16
Csnk2a2
Sema4a
Bicd2
Mpzl2
Zbtb1
Hspa1b
Nqo2
Emcn
Htra3
Rhbdf2
Styk1
Usp38
Pcyox1
Psmb9
Ankrd35
Zcchc6
Dsg1a
Mid2
Gpx4
Plcl2
Nedd9
Hebp1
Rpl3l
Ankrd17
Xpnpep1
4-Sep
Ghitm
Zfand6
Scyl2
Elac1
Fam83g
Ifit1
Wipf2
Ctnnd1
Lrrc28
Ralgapb

Ep300
Gne
Pfkp
Nit2
Nectin4
Ubr5
Lypd3
Epha4
Gm10336
Ece1
Hectd1
Bicdl2
Stk26
Pkp3
Hook1
N4bp2
Grasp
Atg10
1110051M20Rik
Prelid1
Trim21
Gapvd1
Cast
Capn10
B4galnt4
Yod1
Eno1b
Samd10
Actr8
Shroom3
Fbrs
H60c
Fut2
Epha1
Ppp4r3b
Bola3
Aldh9a1
Il4ra
Rhbdl2
Gatc
Tbc1d14

Bst1
Asb14
Dpm3
Gm13710
Lrrc8e
Pou6f1
Foxo3
Cdpf1
Adgrg7
Alox12
Upf1
Traf6
Zcrb1
Zmiz1
Gpr34
Slc25a19
Gipc1
Gstp2
Cox7b
Camsap2
Rad52
Col18a1
Oaz1-ps
Dmap1
Ppp6r3
Tmem45a
Pomgnt2
Eid2b
Cnot1
Jag1
Smim10l1
Ttc39b
Wdr26
Helz2
Mib1
Piwil2
Stat1
Slurp1
Pik3cd
Gpaa1
Efnb2

Bnc1
Atp11a
Fbxw11
Mgat4a
Iffo1
Fbxw2
Tcea3
Cers4
Prkaa2
Ppp1r1a
Strn
Il1rn
Pmepa1
Washc1
Aebp2
Fam210b
Abcb4
Peli1
Gcsh
Yjefn3
Cnpy3
Efl1
Fam83b
Cisd3
Tfg
Mab21l3
Dnajc27
Cd244
Liph
Erc1
Pqlc1
Cited4
Golga1
Ehbp1
Rhbg
Cds2
Myo6
Csrnp1
Adam1a
Col11a2
Asb11

Sh3bp5l
Cx3cr1
Ttc33
Gm8909
Rilpl1
Dsc3
Pdgfc
Kcnn1
Nlrc5
Zfp92
3-Mar
Ephb3
Crot
Hnrnpul2
Mlkl
Rassf3
Rwdd3
Gjb3
Snn
Aadacl2
Zadh2
Serinc5
Pml
Cyth1
Def8
Dhcr7
Slc9a3r2
Ube2d-ps
Itsn2
Serpinb5
Bcap31
Sh3gl1
Tmem154
Nkpd1
Acyp2
